# rTMS-induced motor cortex activation drives neural network tissueoid mediated spinal motor neural pathway reconstruction

**DOI:** 10.7150/thno.117789

**Published:** 2026-01-01

**Authors:** Jing Xu, Yue Yang, Zhen Chen, Jia-Lin Liu, Xiang-Yu Liu, Ming-Yu Lv, Yu-Jian Lin, Jia-Wei Sun, Xiang Zeng, Yuan-Huan Ma, Ge Li, Yi-Nan Guo, Shang-Bin Yang, Miao Tian, Yu Cheng, Rui Liu, Ling Zhang, Xing Li, Xiang Zhou, Yuan-Shan Zeng, Ying Ding, Bi-Qin Lai

**Affiliations:** 1 Center for Stem Cell Biology and Tissue Engineering, Key Laboratory for Stem Cells and Tissue Engineering Ministry of Education, Department of Histoembryology and Cell Biology, Zhongshan School of Medicine, Sun Yat-sen University, Guangzhou, China.; 2 Guangdong Provincial Key Laboratory of Brain Function and Disease, Zhongshan School of Medicine, Sun Yat-sen University, Guangzhou, China.; 3 Co-innovation Center of Neuroregeneration, Nantong University, Nantong, China.; 4 Institute of Spinal Cord Injury, Sun Yat-sen University, Guangzhou, China.; 5 Lab of Stem Cell Biology and Innovative Research of Chinese Medicine; National Institute for Stem Cell Clinical Research, Guangdong Provincial Hospital of Chinese Medicine/The Second Affiliated Hospital of Guangzhou University of Chinese Medicine, Guangzhou, 510120, China.; 6 Guangzhou Institute of Clinical Medicine, Guangzhou First People's Hospital, School of Medicine, South China University of Technology, Guangzhou 510180, China.; 7 Guangdong Provincial Key Laboratory of Pathogenesis, Targeted Prevention and Treatment of Heart Disease, Guangdong Provincial People's Hospital, Southern Medical University, Guangzhou, 510100, China.; 8 Department of Geriatrics, The First Affiliated Hospital, Sun Yat-sen University, Guangzhou, 510080, China.; 9 State Key Laboratory of Dampness Syndrome of Chinese Medicine, Department of Orthopedic Surgery, The Second Affiliated Hospital of Guangzhou University of Chinese Medicine, Guangzhou, China.; 10 Department of Spine Surgery, Center for Orthopedic Surgery, The Third Affiliated Hospital, Southern Medical University, 183 Zhongshan Avenue West, Guangzhou, 510515, China.; 11 Department of Neurosurgery, The First Affiliated Hospital of Guangzhou University of Chinese Medicine, Guangzhou, 510405, China.

**Keywords:** spinal cord injury, rTMS, neural network tissueoid, neural regeneration, motor pathway reconstruction

## Abstract

**Rationale:** The integration of biological and physical interventions represents a promising therapeutic strategy for spinal cord injury (SCI), offering a novel approach to restore disrupted motor pathways. This study investigates whether repetitive transcranial magnetic stimulation (rTMS) can prevent cerebral neuroapoptosis and promote the regeneration and integration of brain-derived nerve fibers with neural network tissueoids (NNToids) following SCI.

**Methods:** Neural stem cell-derived NNToids were transplanted into rats with complete SCI and simultaneously treated with 10 Hz rTMS. Neuroinflammatory responses, neuroapoptosis, neuronal activation, and axonal regeneration were systematically evaluated using transcriptomic sequencing, histological validation, Western blotting, and neural tract tracing. The responsiveness of NNToids to 10 Hz rTMS in facilitating motor neural pathway reconstruction was also assessed.

**Results:** 10 Hz rTMS significantly enhanced cFOS expression in layer V pyramidal neurons of the sensorimotor cortex (SMC), markedly reduced microglial activation and neuroapoptosis, and upregulated the expression of mitochondrial-related protein TOM20, axonal regeneration marker p-S6, and synaptic plasticity-associated protein Arc in SMC neurons. NNToids facilitated the ingrowth of corticospinal tract (CST) and 5-hydroxytryptamine (5-HT) - positive nerve fibers into the transplantation site. Retrograde PRV tracing demonstrated that 10 Hz rTMS enhanced the capacity of NNToid neurons to relay CST and 5-HT signals to hindlimb motor neurons. Functional assessments and cortical motor evoked potentials confirmed that the rTMS-NNToid combination improved the transmission of motor-related neural signals to the hindlimbs. Histological analysis further demonstrated that activated NNToid neurons exhibited increased expression of N-methyl-D-aspartate receptors (NMDAR) and formed more synaptic connections with vGluT-positive axon terminals.

**Conclusion:** These findings demonstrate that rTMS mitigates motor cortex inflammation, promotes the regeneration and integration of brain-derived nerve fibers with NNToid neurons, thereby establishing a foundation for motor function recovery. Moreover, the study identifies the mechanism through which NNToid neurons mediate motor neural pathway reconstruction under rTMS modulation. Although based on a rat model, this work provides a promising framework for future biophysical therapies that combine patient-derived autologous iPSC-based NNToids with non-invasive brain stimulation.

## Introduction

Complete spinal cord injury (SCI) severs the neural pathways between the brain and the body 1. The formation of cavities and scar tissues at the injury site hinders the reconstruction of these pathways 2,3. Moreover, the persistent neuroinflammatory microenvironment may trigger progressive neurodegeneration 4, neuronal apoptosis 5, and muscle atrophy 6, thereby significantly limiting the effectiveness of motor recovery interventions such as brain-computer interfaces and spinal epidural stimulation. A growing body of evidence indicates that neural regeneration and pathway reconstruction are essential structural prerequisites for functional recovery after complete SCI 3,7-9. However, a major challenge remains in optimizing the regeneration of descending axonal tracts and while simultaneously providing relay neurons at the injury site to reconstruct these disrupted neural pathways.

Repetitive transcranial magnetic stimulation (rTMS), a non-invasive neuro-modulation technique that modulates cortical excitability 10, may activate regeneration-related genes and facilitate the reconstruction of descending motor pathway. Among various stimulation paradigms tested in both experimental and clinical settings, 10 Hz rTMS has emerged as a promising candidate for SCI repair 11-13. Its moderate frequency and minimal side effects make it suitable for long-term therapeutic applications. However, most of these studies have been conducted in models of incomplete SCI 14-16, where residual neural pathways may still respond to rTMS modulation.

In the context of complete SCI, even if rTMS promotes brain-derived neuroregeneration 17,18, the absence of interneurons at the injury site—capable of forming synapses with brain-derived neural fibers—may lead to retraction of the regenerated fibers 19. Therefore, a critical challenge in treating complete SCI is replacing pathological cavities and scar tissue with functional neural tissue. This is essential to enable brain-derived neural fibers to locate target neurons for synaptic integration and neural information transmission.

The therapeutic potential of neural stem cell (NSC) transplantation is constrained by two major limitations: the difficulty in directing stem cell differentiation and the delayed neuronal maturation following differentiation 1,20. Although endogenous NSCs activated after SCI 21 can differentiate into new neurons, their slow maturation delays functional motor recovery. Directed differentiation of NSCs into neural network tissues or spinal cord-like tissues via tissue engineering strategies can accelerate neuronal maturation. Our group and others have demonstrated that such engineered neural tissues can rapidly replace necrotic spinal cord tissue, actively modulate the injury microenvironment, and promote host neural fibers ingrowth into the transplantation site 22-25. However, simple transplantation of engineered neural tissues alone cannot promptly reverse brain neurodegeneration or overcome the intrinsic inhibition of brain-derived nerve fiber regeneration after SCI 8,9. Unless brain-derived neural fibers achieve functional integration with transplanted neurons and establish complete neural signal relays, true functional repair of brain-controlled motor pathways cannot be achieved.

Currently, few therapeutic strategies for SCI integrate rTMS with engineered neural tissue transplantation. The mechanism underlying the synergistic effects of this combination remains poorly understood. Based on this gap, we developed a NSC-derived neural network tissueoid (NNToid) enriched with excitatory neurons for SCI repair. We investigated how rTMS activates the motor cortex, modulates NNToid function to relay brain signals, and reconstructs the spinal cord motor pathway. This work aims to advance integrated strategies that combine stem cell-based tissue engineering with non-invasive neuromodulation for central nervous system (CNS) injury repair.

## Materials and Methods

### Construction of Neural Network-like Toid (NNToid)

NSCs were extracted from the hippocampus of 1-day-old GFP transgenic SD neonatal rats (Osaka University, Osaka, Japan). Following the removal of the hippocampal tissue, the outer membrane is peeled and subsequently dissociated in D-Hanks solution. After centrifugation and supernatant removal, NSCs were gently resuspended and cultured in Dulbecco's modified Eagle's medium (DMEM)/F12 (Hyclone, Logan, UT, USA) added with 10 μg/mL B27 (Life Technologies, Gaithersburg, MD, USA) and 20 ng/mL basic fibroblast growth factor (bFGF, Life Technologies, Gaithersburg, MD, USA). The cells were cultured in incubator at 37 ℃ and 5% CO_2_ with suspension state. After 4 days, NSCs were infected with adeno-associated virus (AAV) vector carrying *NT-3* (MOI = 100) and *TrkC* (MOI = 100). Subsequently, the cells were seeded onto collagen sponge scaffolds that had been pre-treated with 0.01% L-type polylysine (PLL) to enhance cell adhesion. The cells were then induced to differentiate on the scaffolds for 14 days, forming NNToids (2 mm in height and 3 mm in diameter), which were subsequently transplanted into the injury site.

### Experimental animals

All animal experiments were conducted in accordance with the guidelines for the Animal Care and Use Committee of Sun Yat-sen University (SYSU-IACUC-2024-B0100). All 220-250 g female SD rats selected in this experiment were provided by the Laboratory Animal Center of East Campus of Sun Yat-sen University. A total of 6 groups were set up in this experiment, namely SCI (given T10 complete SCI), Sham-rTMS (SCI + sham rTMS), rTMS (SCI + rTMS), Sham-NNToid (SCI + sham rTMS + NNToid), rTMS-NNToid (SCI + rTMS + NNToid) and Normal (Healthy rats) group. The Sham-rTMS referred to the sham stimulation with the transmitter coil perpendicular to the skull under same parameters as 10 Hz rTMS. The rats were given cyclosporine injections (5 mg/mL; Catalent Germany Eberbach GmbH; Eberbach, Germany) 3 days before the surgery and every day after surgery.

### Spinal cord transection modeling and transplantation

All surgical procedures were conducted under aseptic conditions in a designated animal operating room. As previously 26, SD rats were anesthetized via intraperitoneal injection of 1% sodium pentobarbital (3.5 μL/g body weight). Following anesthesia, a midline incision was made using a scalpel to expose the vertebral laminae. The dorsal lamina of the T9-T10 vertebrae was carefully removed using a bone rongeur to expose the underlying spinal cord. A complete spinal cord transection was performed at the T10 spinal segment using a 2 mm vertical cut along the transverse plane, resulting in the removal of a cylindrical segment of the spinal cord. Complete spinal cord transection was confirmed by the absence of Cortical Motor Evoked Potentials (CMEPs) of hindlimbs recorded immediately post-operatively. Rats that satisfied the criteria were incorporated into the experiment. When the hemostasis was sufficient, NNToid were implanted in Sham-NNToid and rTMS-NNToid animals. No scaffold was implanted in the SCI and Sham-rTMS group. After that, 4-0 surgical sutures were used to stitch the soft tissues layer by layer. All surgical procedures were carried out in a sterile environment. Postoperatively, the rats were given daily intramuscular administration of penicillin at a dose of 50,000 units/kg, continuous injection of the analgesic buprenorphine (50 μg/kg) for 7 days, and urination assistance twice a day.

### Transcranial magnetic stimulation therapy

Rats in the rTMS and rTMS-NNToid groups received treatment on the third postoperative day using a specialized round coil (radius: 32 mm, model Y064) connected to a MS therapy device (Yiruide, Wuhan, China). The rTMS stimulation consisted of 30 pulses (10 Hz), which was repeated 66 times for a total of 1980 pulses. The stimulation output intensity (23%) was set as 90% of the movement threshold (MT) 18,27. The therapy (stimulation time 3 s, interval 5 s, total treatment time 8 mins 48 s) was performed 5 times a week for 8 weeks. During the implementation of rTMS on rats, the stimulation site of sensorimotor cortex (SMC) was identified by determining whether action potentials were evoked in the hindlimbs. The stimulation coil was positioned precisely over the cranial midline to ensure optimal targeting of the underlying neural structures. Subsequently, the activation of neurons in the SMC region was further determined through histological detection to confirm that the neurons had been widely stimulated. The identical conditions were used to falsely stimulate the Sham-rTMS and Sham-NNToid groups using a transmitting coil positioned perpendicular to the skull. Normal rats (*n* = 32) were selected to measure the average MT. The ground electrode was attached on the back of rats, the stimulation coil was positioned above the middle of the head, and recording electrode captured the recorded potential of the anterior tibialis muscle. Under anesthesia, the CMEP of the rats was measured, beginning with the minimum stimulation intensity that could cause dorsiflexion of the ankle joint. MT was determined as the lowest intensity at which, when the muscle exhibited a slight contraction, the amplitude of 5 CMEPs was not less than 100 μV.

### Behavioral test

As previously 28, the motor function of the rats' hindlimbs was assessed weekly post-surgery using an open-field locomotion test. Evaluations were conducted in a double-blind manner and scored according to the Basso-Beattie-Bresnahan (BBB) scale 29, which primarily focuses on voluntary hindlimb movements and weight-bearing capacity. At the 8th week post-operation, a comprehensive evaluation of motor function and coordinated hindlimb movements were performed using the modified inclined-grid climbing test 30 and mirror experiment. Each rat was given 3 chances to climb the grid with a 45° inclination in the inclined-grid climbing test. The rats were randomly selected from groups for behavioral statistical analysis. The BBB scores were calculated as the average of the scores assigned to the left and right hind limbs. And during 3 crawling experiments per rat, the number of unilateral hindlimb grasps was quantified for statistical analyses. Both the BBB scores and the number of grasps were independently evaluated by two individuals. In the double-blind assessment, the two independent raters were aware only of the rat identification numbers and remained blinded to the experimental group assignments. The scores from the two independent raters were computed the mean scores performed group-based statistical analyses. And the rat on a flat grid can be more intuitively observed movements in the mirror experiment.

### Cortical Motor Evoked Potentials (CMEPs)

At 8th week after surgery, the experimental animals were anesthetized and assessed for CMEPs using CCY1 magnetic stimulation apparatus. During the procedure, the magnetic field generated by the stimulation coil penetrated the skull. The output intensity was set at the maximum stimulation level (100%). Electrophysiological potentials were collected from the tibialis anterior muscle following transcranial stimulation. The mean latency and amplitude of the evoked responses were measured and analyzed. For quantitative analysis, 5 rats were randomly selected from each group. For each rat, 10 qualified response waves were meticulously chosen. Specifically, 5 response waves were separately selected from each of the left and right hindlimbs. These qualified response waves had distinct onsets (O), peaks (P), and valleys (V), which clearly indicated the positions of the waveforms. Subsequently, the average values of the latency and amplitude for each individual rat were calculated. In the magnetic stimulator system, the 10 waveforms of each rat were accurately fitted into one representative waveform.

### Biotinylated Dextran Amine (BDA) tracing in the SMC region

At 8 weeks after SCI, the rats were selected for anterograde BDA tracing. The surgical procedure was carried out on a stereotactic apparatus for the brain. Following anesthesia, the head's skin and fascia were removed to reveal the skull. Then, the skull above the SMC region was then removed to fully expose the brain tissue. Two circular cranial windows, each with a diameter of 5 mm, were created at symmetrical locations. A 10% BDA-1000 solution (Invitrogen, MA, USA) was administered via stereotaxic injection into SMC region, carefully avoiding vascular structures to minimize tissue damage and ensure precise targeting. In each cortex, 9 equally spaced injection sites were chosen. Each injection site received 0.5 µL of solution, resulting in a bilateral volume of 9 µL. The perfusions were performed 1 mm below the cortical surface, and the needle was kept in place for 2 mins following the injection. The head fascia and skin were sutured post injection. The animals were perfused for further analysis 2 weeks post injection.

### Perfusion and tissue preparation

Following euthanasia via anesthetic overdose, the thoracic cavity of the rat was surgically opened to expose the heart. A perfusion tube was inserted into the aorta through the left ventricle. Systemic perfusion was initiated with a vascular rinse solution (0.09 g/mL NaCl, 0.02 ‰ sodium nitrite) to remove residual blood. Subsequently, the brain and spinal cord tissues were carefully dissected and immersed in 4% paraformaldehyde (PFA, Merck, Darmstadt, Germany) at 4 °C for 24 hours for fixation. Following fixation, the tissues were subjected to gradient dehydration using increasing concentrations of sucrose. The dehydrated tissues were processed for embedding in Neg-50^TM^ water-soluble frozen section medium (Epredia, Michigan, USA) and subsequently sectioned into 25 μm thick slices using a cryostat (Thermo Scientific, MA, USA). The sections were stored at -30 °C until further use.

### Western blot

Proteins were extracted from SMC tissue and spinal cord tissue at the rostral of injury site. A polyacrylamide gel was prepared using the commercial kit (CWBIO, Beijing, China), and the processed protein samples were loaded into the wells of the gel. Following electrophoresis, proteins were wetly transferred from the gel to a polyvinylidene fluoride (PVDF) membrane. The membrane was blocked with a blocking solution at room temperature for 1 hour. Subsequently, the membrane was then exposed to the primary antibody at 4 °C for 24 h. After 3 washes with Tris-buffered saline containing Tween-20 (TBST), the membrane was incubated with the secondary antibody at room temperature for 1.5 hours. Protein bands were visualized using an enhanced chemiluminescence (ECL) immunoassay kit in ChemiDoc Touch system (Bio-Rad, CA, USA).

### mRNA sequencing

The SMC region tissue and spinal cord tissue at the rostral of the injured area were harvested from 3 rats per group, including the Normal, Sham-rTMS, and rTMS groups. The collected tissues were promptly immersed in liquid nitrogen within cryogenic storage tubes to ensure rapid freezing. For RNA-Seq analysis, samples were analyzed to evaluate transcriptomic profiles by Genergy Bio-Technology Co., Ltd. StringTie was used to evaluate the initial sequence numbers for identified genes of the samples from Normal, Sham-rTMS and rTMS groups. A corrected P value ≤ 0.05 and |log2(fold change)| ≥ 1 was applied as the criterion for identifying differentially expressed genes, to ensure robust and statistically rigorous results.

### Protein-protein interaction (PPI) analysis and network construction

For further analysis, we retrieved genes associated with canonical brain regeneration and synaptic plasticity pathways, along with differentially expressed genes (DEGs) in neural activation and regeneration pathways. Using the STRING database, a PPI network was constructed with the key module genes, employing a high-confidence minimum interaction score of 0.7 and default settings for all other parameters. The resulting PPI network was subsequently visualized and analyzed in Cytoscape (v3.10.3) using a topology-based method with degree centrality.

### Immunofluorescence analysis

After equilibrating the tissue sections to room temperature, residual impurities were removed by rinsing with 0.01 mol/L phosphate-buffered saline (PBS). To minimize non-specific binding and reduce background staining, the sections were incubated with 10% goat serum at 37 °C for 30 mins. Subsequently, the sections were treated with specific primary antibodies and incubated at 4 °C overnight. The following day, unbound primary antibodies were removed by washing with PBS, and the sections were incubated with appropriate secondary antibodies at 37 °C for 1 hour. After another PBS wash, nuclear staining was performed using Hoechst staining solution for 15 mins at room temperature. Finally, the sections were rinsed with PBS and mounted with 80% glycerol for microscopic analysis. Imaging was performed by confocal microscopy (Dragonfly, Andor Technology, Belfast, UK). Table S1 provides an overview of antibodies used for immunofluorescence.

### Hematoxylin and Eosin (HE) staining

Tissue was harvested and subsequently processed into frozen slices. Prior to staining the tissue, the slices were initially hydrated by the gradient of ethanol. Subsequently, the slices were soaked in hematoxylin for 5 mins and rinsed with distilled water. Next, the slices were immersed in 0.5% eosin solution for 10 mins, and rinsed with distilled water. Then the dehydration was performed with gradient ethanol. To finish tissue permeabilization, the slices were immersed in xylene solution for 10 mins and repeated twice. Finally, neutral gum was used for mounting slices.

### Culture of SMC neurons

The neurons were obtained from the SMC region of the cerebral cortex of 0 days-old newborn SD rats. The SMC tissues isolated from the cerebral cortex were dissociated in the neuronal protective fluid (D-Hanks solution containing 1% glucose). Then, the solution containing 0.125% pancreatic enzyme and 0.1% DNAnase were used to digest tissues at 37 ℃ for 10 min, and the suspension centrifuged to obtain cell precipitation after removing supernatant. The precipitate was washed twice with neuroprotective solution. Single cell suspension was obtained by filtering with cell sieve (Millipore, Bedford, MA, USA). The cells were transferred to a 24-well plate containing glass coverslips pre-coated with laminin (20 μg/mL) for culture. The cell medium was composed of neurobasal medium (Gibco, Waltham, USA) supplemented with B27 (1×, Gibco, Waltham, USA) and 0.5 mM glutamine (Sigma, Madrid, Spain).

### Pseudorabies virus (PRV) tracing

Following 8 weeks of therapy, the animals in the rTMS-NNToid and Sham-NNToid groups were anesthetized with isoflurane (1.2%) and injected with the PRV (PRV-CAG-mRFP, BrainVTA, Wuhan, China). A longitudinal incision was performed in the skin covering the gluteal area and the superior part of the thigh using a scalpel. To optimize exposure of the sciatic nerve and minimize bleeding, the muscle was bluntly split along the course of its fibers. A glass minute hand was then used to isolate the sciatic nerve, and the viral solution was administered using a glass micropipette. Five injection sites were selected along each sciatic nerve, and 0.3 µL of virus was injected at each site, with a 1-min interval between injections. Each animal was administered 3 μL viral solution. The animals were sacrificed 7 days later.

### Morphological quantification

To assess inflammatory and apoptotic responses in SMC region, we measured the number of microglial cells and the proportion of cleaved caspase 3 (CC3) and neuronal nuclear antigen (NeuN) positive neurons in the field manually. The phosphorylated S6 (p-S6) activity in various cortical neurons was determined by the fluorescence area ratio in each layer using Image J. And the total fluorescence quantification of p-S6 in SMC region was the cumulative value of layer I-VI. To quantify cFOS activation in the SMC region in vivo, we measured the number of cFOS^+^NeuN^+^ neurons in the field manually. In addition, the fluorescence intensity of activity regulated cytoskeletal (Arc) and translocase of outer mitochondrial membrane 20 (TOM20) is detected using Image J. To quantify the three differentiations of GFP-NNToid in vivo, we manually calculated the number ratio of Map2^+^GFP^+^/GFP^+^, MBP^+^GFP^+^/GFP^+^, and GFAP^+^GFP^+^/GFP^+^ cells, respectively. We randomly selected 2 mm × 2 mm regions in the rostral, central, and caudal of injury/graft (I/G) sites, and calculated the fluorescence area ratio of NF^+^, GAP43^+^ neural fibers using ImageJ to assess nerve fiber regeneration. For the analysis of HE staining, the Image J was used to calculated the ratio of the area of cavities within a 1.25 mm lateral distance and 1 mm in height from the midline in the I/G site. The excitation of raphe nucleus (RN) was evaluated by manually measuring the number of cFOS^+^ cells in RN region. What's more, the connection between corticospinal tract (CST) and 5-hydroxytryptamine (5-HT) energy neural fibers was evaluated by the number of BDA^+^ puncta on 5-HT^+^ neurons in the RN region. The regeneration of CST was measured by the BDA^+^ neural fibers (≥ 10 μm in length) in the chosen fields in different regions of the spinal cord. And the regenerated 5-HT neural fibers were detected by the fluorescence intensity of 5-HT in the selected fields using Image J. For the quantitative analysis of PRV^+^ neurons, the number of PRV^+^ neurons within a 20 × field was calculated in the SMC region, mesencephalon, C4 segment, and I/G site. And the total population of PRV^+^ neurons was calculated in complete histological cross-sections of the L4 spinal cord segment. The fluorescence intensity ratio of vesicle glutamate transporters 1 (vGluT1)/GFP, vesicle glutamate transporters 2 (vGluT2)/GFP, NMDAR/Map2 and GS/GFAP in the selected fields of the I/G site was also measured by ImageJ to determine the response ability of GFP-NNToid to excitatory nerve information.

### Data analysis

All statistical analyses were performed using GraphPad Prism software. Data are presented as mean ± standard deviation (SD). Shapiro-Wilk normality testing was used to evaluate data distribution. Brown-Forsythe testing was utilized to test the homogeneity of variance. Statistical comparisons between two groups were assessed using an unpaired Student's t-test. For comparisons involving more than two groups with normal distribution, one-way analysis of variance (ANOVA) followed by post hoc tests and Dunnett's test was applied. For data sets that do not conform to the assumption of normality, the intergroup differences were assessed by nonparametric Kruskal-Wallis test, followed by Dunn's post hoc test. Statistical significance was defined as a p-value less than 0.05 in all analyses.

## Results

### The effects of rTMS on neural activation and synaptic plasticity in the SMC region

**Figure 1:**
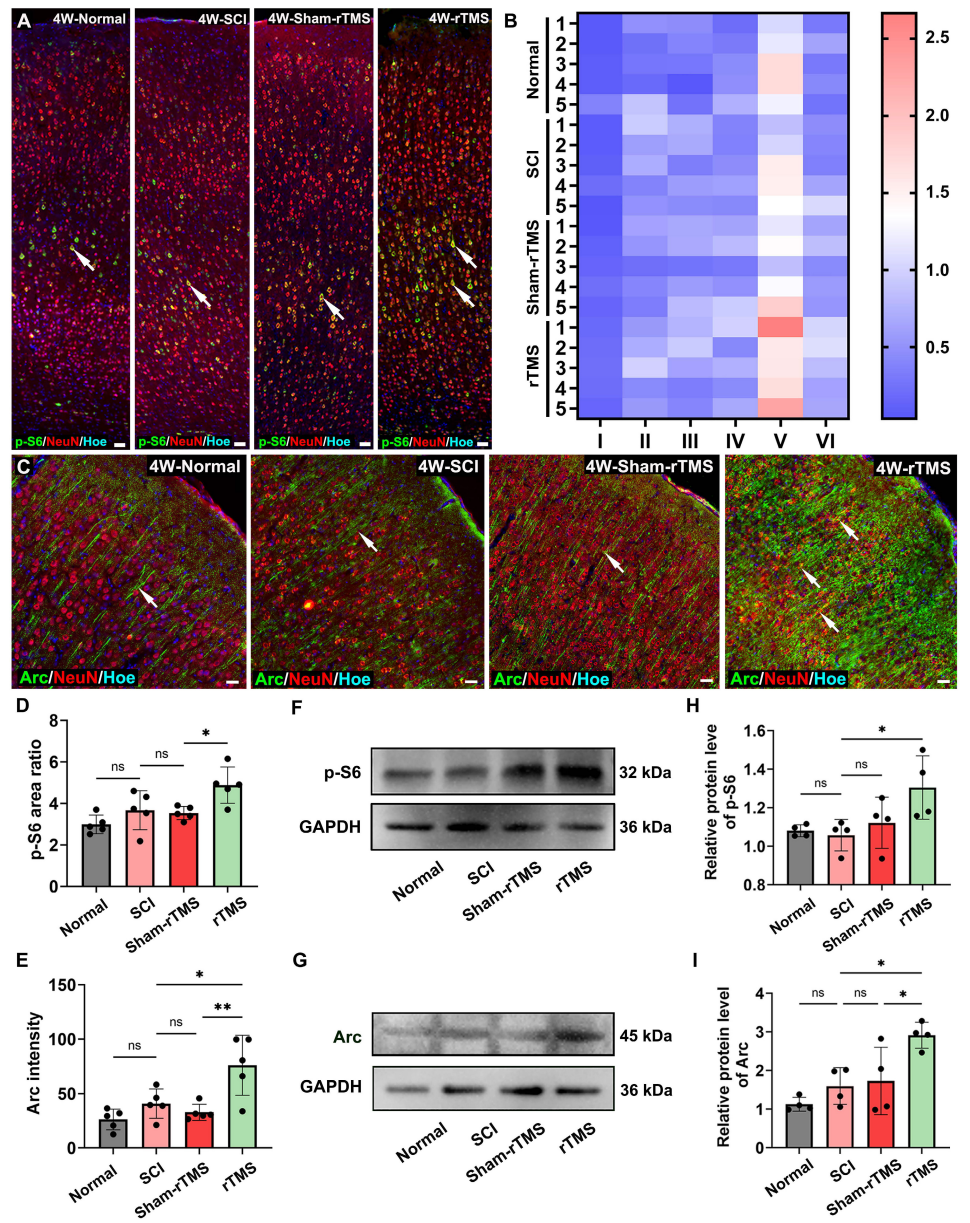
** rTMS promoted activation and plasticity of SMC neurons.** (A) The immunofluorescence staining of p-S6 (arrows) in SMC regions. (B) Visualization of average fluorescence area ratio of p-S6 in I-VI layers of SMC region. (C) Immunofluorescence staining of Arc (arrows) in SMC regions. (D-E) Quantitative analysis of p-S6 (cumulative fluorescence area ratio of I-VI layers) and Arc fluorescence intensity 4 weeks post SCI. *n* = 5. (F-G) WB analysis showed the expression of p-S6 and Arc protein 4 weeks post SCI in each group. (H-I) Quantitative analysis of p-S6 and Arc proteins. *n* = 4. WB: western blot. Scale bars = 50 μm in A; 20 μm in C. **p < 0.01, *p < 0.05, ns: non-significant. Data showed mean ± SD.

Given that the cortical neuronal silencing occurred following SCI, we first assessed the efficacy of rTMS in restoring cortical neuronal activation. The results showed that the 10 Hz rTMS, which is commonly used in clinical, could obviously activate the SMC neurons (Figure S1). Following the establishing the complete T10 transection model in rats (Figure S2A-D), 10 Hz rTMS was administered at the stimulus intensity of 23% (Figure S2E). Transcriptomic sequencing results (Figure S2F) further confirmed that genes associated with neuronal activation and regeneration, were significantly upregulated in the rTMS group. To further explore the intrinsic regeneration regulatory mechanism of rTMS on SMC neurons, we identified two downstream proteins related to neural activation, regeneration and synaptic plasticity (Figure S2G) through the PPI. Primarily, we detected the fluorescence expression of p-S6, a marker protein closely related to neuronal protein synthesis and axonal regeneration. Figure 1A showed that p-S6 was activated to various degrees in SMC neurons across all groups. Notably, in the rTMS group, the p-S6 expression was considerably higher in layer V pyramidal neurons (Figure 1B). The Arc associated protein plays a crucial role in synaptic plasticity 31. Arc is mainly expressed in excitatory pyramidal neurons, and the fluorescence signal is obvious in the cell bodies and proximal dendrites (Figure 1C). The fluorescence expression and protein levels of p-S6 and Arc in the rTMS group were elevated notably compared with those in the SCI and Sham-rTMS groups (Figure 1D-I).

### rTMS exerts neuroprotective effects and promotes nerve fiber regeneration

Following complete SCI, retrograde dysregulation of the cortical neuroimmune microenvironment was detected in the SMC region. Immunofluorescence analysis revealed rTMS treatment elicited neuroprotective effect in the SMC region. Compared to SCI and Sham-rTMS controls, the rTMS group exhibited progressive reductions in the number of microglia and CC3 expression (Figure S3A-D). Western blot quantification confirmed these findings, showing duration-dependent attenuation of IBA-1 (Figure S3E, G) and CC3 (Figure S3F, H) protein level. The transcriptome sequencing results (Figure S3I, J) also showed that anti-inflammatory and anti-apoptotic genes in the SMC region of the rTMS group were upregulated, while pro-inflammatory and pro-apoptotic genes were downregulated. The results showed that rTMS intervention post-SCI is crucial for maintaining immune homeostasis in SMC neurons. Additionally, compared with the SCI group (Figure S4A) and the Sham-rTMS group (Figure S4B), 4 weeks-rTMS treatment significantly increased the fluorescence area ratio of GAP43^+^ and neurofilaments (NF)^+^ neural fibers in both the rostral and central lesion areas (Figure S4C-D). Collectively, these findings indicated that initiating rTMS could prevent neurodegenerative progression and promote nerve fiber regeneration.

### Construction of NNToid in vitro

**Figure 2:**
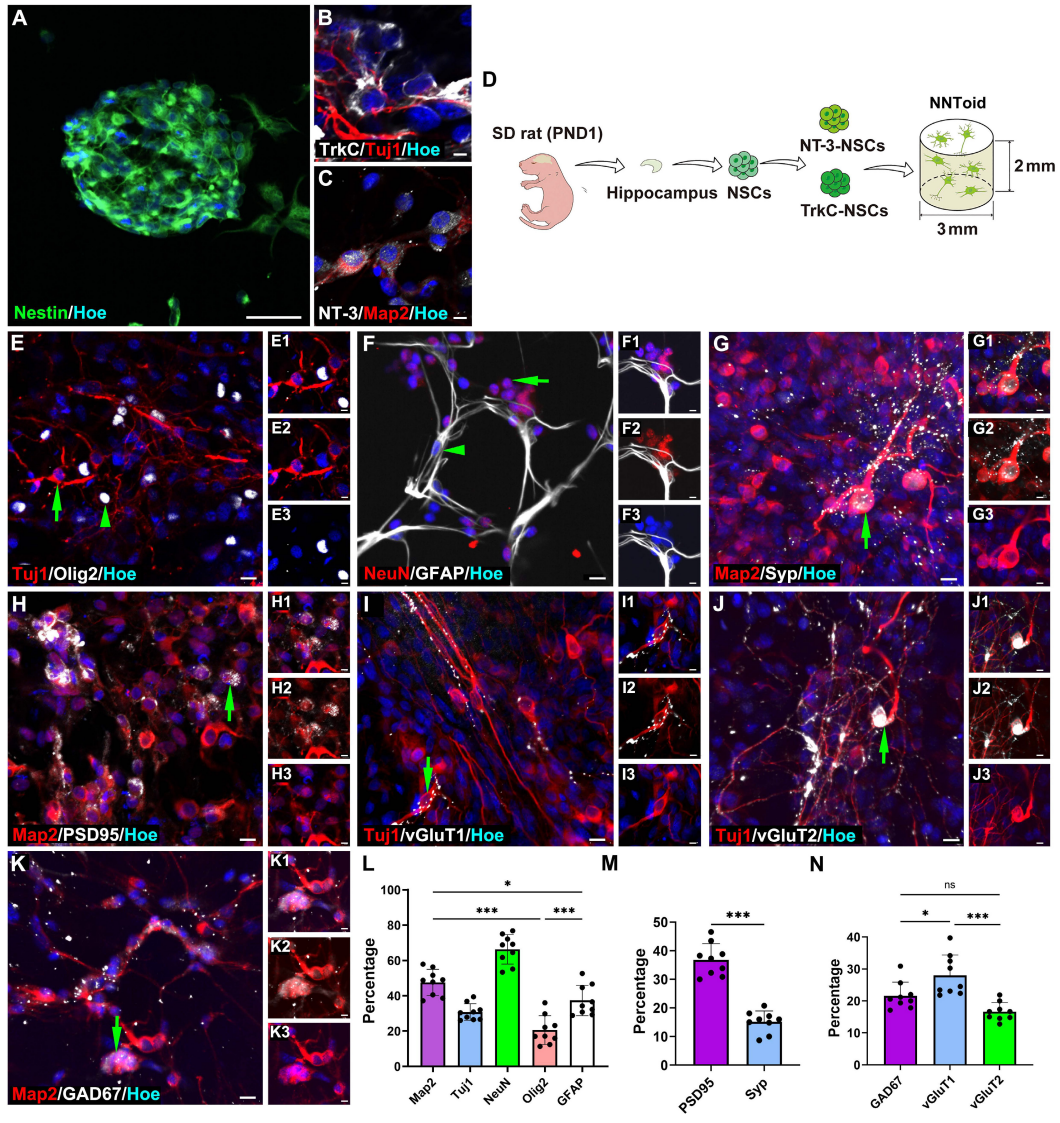
** The fabrication and characterization characteristics of NNToid.** (A) Nestin^+^ cells spontaneously formed a neurosphere. NNToid-derived neurons both expressed NT-3 (B) and TrkC protein (C). (D) A schematic of NNToid constructional process. PND1: post natural day 1. (E-K) Differentiating phenotypes and surface markers of NSCs on NNToid. (E) NSCs within NNToid differentiated into Tuj1^+^ cells (arrow), Olig2^+^ cells (arrow head), (F) NeuN^+^ cells (arrow) and GFAP^+^ cells (arrow head). Differentiated Map2^+^ neurons (arrows) within NNToid expressed Syp (G), PSD95 (H), excitatory transmitter vGluT1 (I), vGluT2 (J) and inhibitory transmitter GAD67 (K). (L) Differentiation ratio of phenotype cells from NSCs within NNToid. (M, N) Bar Charts showing that the percentage of Syp^+^, PSD95^+^, GAD67^+^, vGluT1^+^ and vGluT2^+^ neurons. *n* = 9. Scale bars = 30 μm in (A), 5 μm in (B-C), E (1-3)-K (1-3); 10 μm in (E-K). ns: non-significant. ***p < 0.001, *p < 0.05. Data showed mean ± SD.

Based on the therapeutic potential of rTMS, we proposed the combination of rTMS with NNToid transplantation as a promising therapeutic strategy for SCI. Subsequently, the in-vitro fabrication of NNToid and the comprehensive characterization of their properties were performed. Hippocampus-derived NSCs were cultured in suspension and formed nestin-positive neurospheres (Figure 2A). These NSCs were subsequently genetically modified to overexpress *NT-3* and *TrkC*, then seeded at a 1:1 ratio onto a collagen sponge scaffold with a diameter of 3 mm and a height of 2 mm. After a defined period of 3D tissue culture, NNToid were successfully generated. The immunostaining analysis revealed that the differentiated neurons within the NNToid maintained the expression of NT-3 or TrkC (Figure 2B, C). The engineered NNToid were scheduled for transplantation into the location of complete spinal cord transection (Figure 2D).

Prior to transplantation, the comprehensive characterization of NNToid were performed to determine identifying the expression of immature neuronal marker β-tubulin III (Tuj1), mature neuronal nuclear antigen (NeuN), oligodendrocyte marker oligodendrocyte precursor transcription factor 2 (Olig2), and astrocyte marker glial fibrillary acidic protein (GFAP). Semi-quantitative analysis showed that neurons represented the predominant cell population within NNToid, with approximately 50% of the cells exhibiting positive staining for microtubule-associated protein 2 (Map2), a mature neuron marker. Furthermore, Tuj1 and NeuN-positive neurons accounted for approximately 30% and 65% of the total cell population, respectively (Figure 2E-G, L). To evaluate the synaptic formation capacity of NNToid-derived neurons, we examined the expression of synaptic markers. Both synaptophysin (Syp) and postsynaptic density protein 95 (PSD95) were prominently expressed in neuronal cell bodies and neurites (Figure 2G-H, M). Further characterization revealed that NNToid-derived neurons expressed both excitatory neurotransmitter (vGluT1, vGluT2) and inhibitory neurotransmitter marker glutamate decarboxylase 67 (GAD67) (Figure 2I-K, N). The relative expression profiles suggested a predominance of excitatory neurons within the NNToid.

### rTMS modulates both neural metabolism and regeneration of SMC neurons following NNToid transplantation

**Figure 3:**
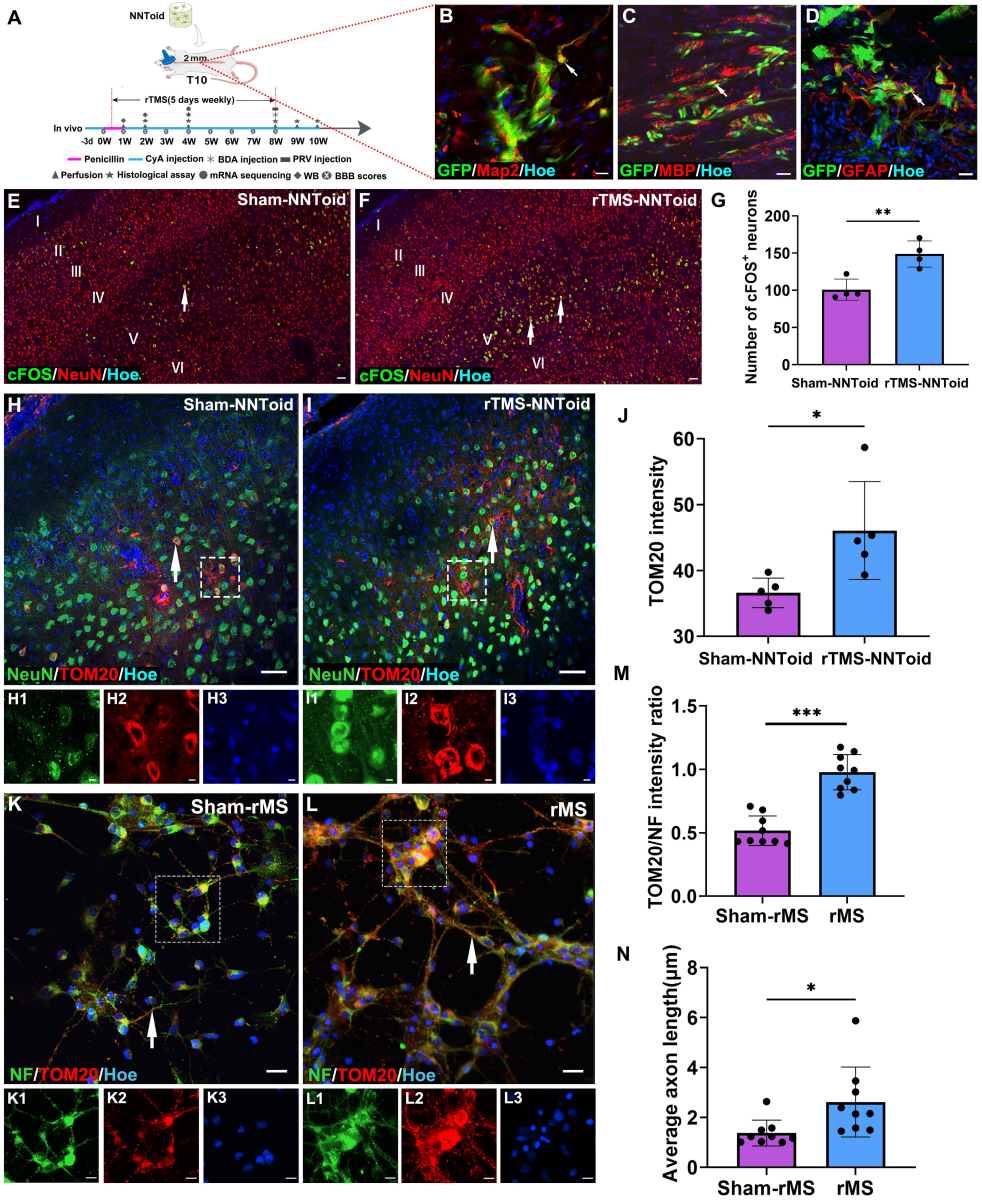
** rTMS enhanced energy metabolism and neurite growth in SMC neurons.** (A) NNToid was transplanted into injured area of SD rats with T10 complete SCI. Some rats were administered with rTMS treatments 72h post SCI. The diagram illustrated the experimental timeline. CyA: cyclosporin, WB: western blot. (B-D) The Map2^+^ (arrows), MBP^+^ (arrows) and GFAP^+^ cells (arrows) derived from GFP^+^ transplanted cells within NNToid in I/G site. I/G: Injury/Graft. (E-F) Representative images demonstrating that enhanced cFOS activity (arrows) in SMC region of rats treated with rTMS-NNToid for 8 weeks, especially the layer V. (G) Quantification of the average number of cFOS^+^ neurons in the fields. *n* = 4. (H-I) Immunofluorescence images showed the expression of TOM20 (arrows) in SMC neurons of Sham-NNToid and rTMS-NNToid groups. (H1-H3, I1-I3) The corresponding local enlarged image from the frame in (H-I). (J) Quantitative immunofluorescence analysis of TOM20 in (H, I). *n* = 5. (K-L) Immunofluorescence detection of TOM20 (arrows) in vitro after Sham-rMS and rMS treatment in SMC neurons. (K1-K3, L1-L3) were the corresponding local enlarged images from the frame in (K-L). (M) Bar chart showing immunofluorescence intensity ratio of TOM20/NF in the Sham-rMS and rMS groups. *n* = 9. (N) Bar chart showing the average neurite length of SMC neurons in the Sham-rMS and rMS groups. *n* = 9. Scale bars = 20 μm in (B-D), 60 μm in (E-F), 40 μm in (H-I), 5 μm in (H1-H3, I1-I3, K1-K3, L1-L3), 20 μm in (K, L). ***p < 0.001, **p < 0.01, *p < 0.05. Data showed mean ± SD.

After identifying NNToid characteristics in vitro, we combined rTMS and NNToid (rTMS-NNToid combination) to treat SCI rats (Figure 3A). Following 8 weeks of rTMS-NNToid combination, GFP-positive cells demonstrated robust survival in both Sham-NNToid and rTMS-NNToid groups, and maintained stable differentiation characteristics (Figure 3B-D). The ratios of differentiation markers Map2 (Figure S5A-B, G), MBP (Figure S5C-D, H), and GFAP (Figure S5E-F, I) showed no significant difference between the Sham-NNToid and rTMS-NNToid groups. However, rTMS-NNToid combination can effectively repopulate the voids in the damaged area. The HE staining results revealed that the area of voids in the rTMS-NNToid group was significantly decreased compared to controls (Figure S6). The cFOS detection suggested that the layer V pyramidal neurons were particularly susceptible to excitation in rTMS-NNToid group (Figure 3E-G). Immunofluorescence quantification showed that the expression level of TOM20 was significantly upregulated in the rTMS-NNToid group compared to the Sham-NNToid group (Figure 3H-J). This indicated an increase in both mitochondrial abundance and energy metabolism levels in SMC neurons, particularly layer V pyramidal neurons, following the combined treatment. To further verify the results in vivo, we cultured SMC-derived neurons from neonatal rats in vitro. After exposure to 10 Hz repetitive magnetic stimulation (rMS), which mimicked the in vivo treatment pattern, TOM20 expression levels were significantly upregulated (Figure 3K-M), and the length of NF-positive neurites was significantly increased (Figure 3N). Meanwhile, rMS enhanced neuronal excitability (Figure S7A-C) and Syp expression (Figure S7D-F). Collectively, these results suggested that rTMS could stimulate the SMC neurons. By enhancing energy metabolism, it might facilitate the outgrowth of their neurites and the formation of synapses.

### The motor function recovery in rats following combined rTMS treatment

**Figure 4:**
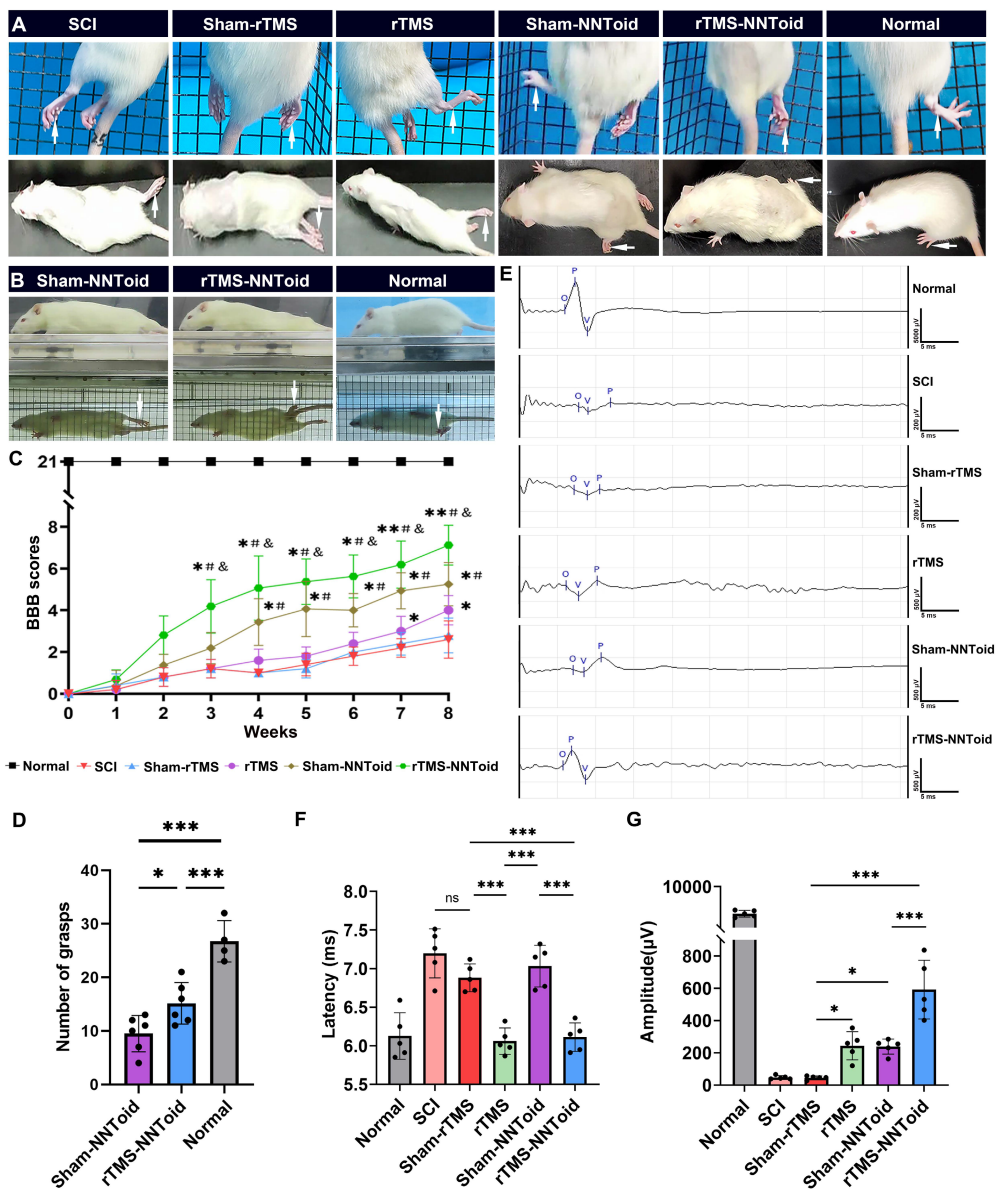
** rTMS-NNToid combination improved motor function and electro-physiological activity.** (A) Representative pictures of the hindlimbs (arrows) locomotor in climbing grid experiment and the open field test. (B) Representative pictures of the hindlimbs (arrows) locomotor in the mirror experiment. (C) Hindlimbs locomotor BBB scores 8 weeks post SCI. *n* = 8 in Normal, Sham-NNToid, rTMS-NNToid groups; *n* = 5 in SCI, Sham-rTMS, rTMS groups. *, #, & showed statistical significance in relation to Sham-rTMS, rTMS and Sham-NNToid group, respectively. (D) This bar chart illustrated the number of grasps performed by the unilateral hind limb of rats during the inclined grid test. *n* = 6 in Sham-NNToid and rTMS-NNToid groups, *n* = 4 in Normal group. (E) Representative images of CMEPs 8 weeks post SCI. (F-G) Quantitative analysis of the latency and amplitude of CMEPs in each group. *n* = 5. 10 response waves were selected from each rat. ***p < 0.001, *p < 0.05, ns: non-significant. Data showed mean ± SD.

Subsequently, we further analyzed hindlimb-motor improvement 8 weeks after surgery. In the grid-climbing test (Figure 4A, Movie S1), SCI and Sham-rTMS groups predominantly exhibited forelimb-dependent locomotion with complete hindlimb paralysis and absence of spontaneous placement reflex. The rTMS and the Sham-NNToid groups showed limited hindlimb movement, characterized by occasional foot placement. Notably, the rTMS-NNToid group demonstrated significant motor improvement, with intermittent toe grasping observed. These findings were corroborated by mirror experiments (Figure 4B, Movie S2). BBB locomotor scores revealed that all rats exhibited complete paralysis (score = 0) at 0 days post SCI. Gradual recovery of motor function was observed across all groups within 2 - 3 weeks. By the 8th week, BBB scores in the SCI and the Sham-rTMS groups reached approximately 3 points, indicating minimal joint movement. The rTMS group showed slight improvement in three-joint mobility, while the Sham-NNToid group exhibited discontinuous foot-dragging locomotion. However, the rTMS-NNToid group demonstrated superior functional recovery (Figure 4C-D, Movie S3), exhibiting continuous foot-dragging with occasional weight-bearing steps. Following complete spinal cord transection, the amplitude and latency of CMEPs reflect the number of regenerating axons and nerve conduction velocity, respectively. Electrophysiological assessment revealed significantly reduced CMEPs amplitude and prolonged latency in the SCI and Sham-rTMS groups post-injury (Figure 4E). The rTMS and Sham-NNToid groups showed moderate improvement in CMEP amplitude, with reduced latency in the rTMS group. Most notably, the rTMS-NNToid group exhibited substantial enhancement in both CMEP amplitude and conduction velocity (Figure 4F, G). Collectively, these findings indicated that the rTMS-NNToid combination significantly enhanced hindlimb motor function recovery and improved neural conduction in rats post SCI.

### rTMS combined with NNToid therapy promotes nerve fiber regeneration

To further elucidate the mechanisms underlying motor function improvement through rTMS-NNToid combination therapy, we conducted a systematic analysis of spinal cord nerve fiber regeneration. As shown in (Figure S8A, B), the rTMS-NNToid group exhibited increased GAP43 and NF double positive fibers in the rostral compared to the Sham-NNToid group, with more fibers growing into the I/G site and synaptic formations with the NNToids (Figure S8C, D). Regional quantification confirmed a significant increase in fiber regeneration in both the rostral (*p* < 0.05) and central (*p* < 0.05) of the injury region compared to Sham-NNToid controls, with no significant difference in the caudal region (Figure S8E).

### rTMS modulates the plasticity of corticospinal tract-controlled motor pathways following NNToid transplantation

**Figure 5:**
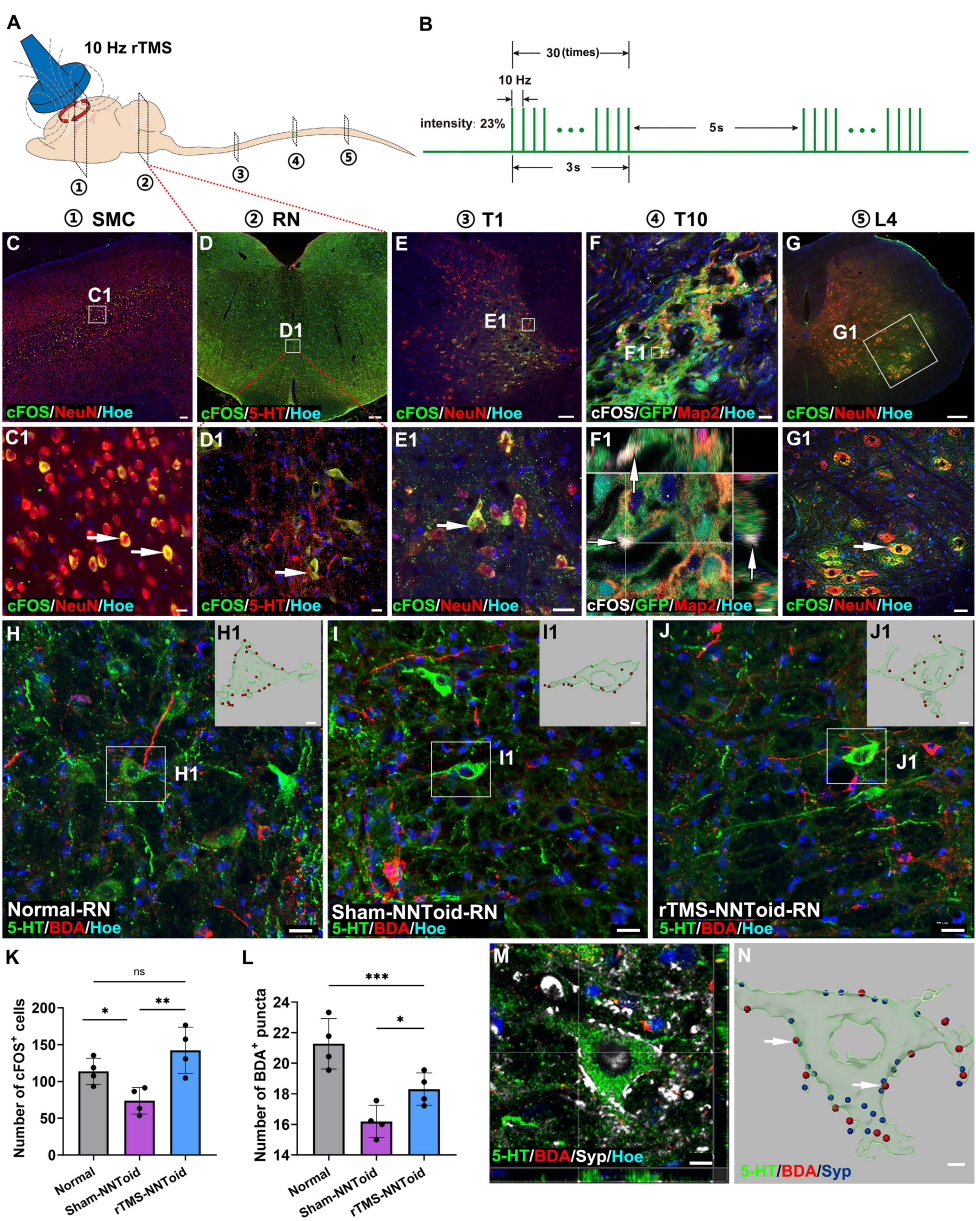
** rTMS activated the brain-spinal nerve pathway.** (A) Schematic diagram of the anatomical locations for detecting the activation status of neurons in the brain-spinal cord pathway in rTMS-NNToid group at 8 weeks post SCI. ①: Somatosensory motor cortex (SMC); ②: Raphe nucleus (RN); ③: Thoracic 1 segment (T1); ④: Thoracic 10 segment (T10); ⑤: Lumbar 4 segment (L4). (B) Pulse diagram of the rTMS. (C-E) Expression of cFOS^+^ excitatory neurons in the SMC region, RN, and T1 of the rTMS-NNToid group. (C1-E1) The magnified areas of the corresponding low-power fields. The arrows indicated cFOS^+^ neurons. (F) Represented field of the I/G site. (F1) Orthogonal image showed the expression of cFOS (arrows) in GFP NNToid. (G) Expression of cFOS^+^ neurons in L4. (G1) The magnified area of the corresponding low-power field. The arrow indicated the cFOS^+^ neurons. (H-J) Interaction between BDA^+^ CST neural fibers and 5-HT^+^ neurons in the RN. (H1-J1) 3D reconstructions of the magnified areas in (H-J). BDA puncta: red, 5-HT^+^ neurons: green. (K) Quantification of cFOS^+^ cells in the RN. *n* = 4. (L) Quantification of BDA^+^ puncta on the surface of 5-HT^+^ neuron in each group. *n* = 4. (M) Synaptic connections formed between 5-HT^+^ neurons and BDA^+^ puncta on the surface of neurons in the RN. (N) 3D reconstruction of M. Arrows indicated the contact between BDA puncta (red) and synaptic puncta (blue) on the surface of 5-HT^+^ neurons (green). I/G: injury/graft. Scale bars = 60 μm in C, 250 μm in D; 20 μm in (C1, D1, E1, F, H-J); 100 μm in E; 2 μm in F1; 150 μm in G; 40 μm in G1; 5 μm in (H1-J1, N); 10 μm in M. ***p < 0.001, **p < 0.01, *p < 0.05, ns: non-significant. Data showed mean ± SD.

We hypothesized that NNToid transplantation combined with sustained rTMS treatment would exert a significant influence on the CST and its descending motor pathway (Figure 5A-B). The cFOS detection after 8 weeks of continuous treatment revealed activation of SMC neurons and 5-HT neurons in the rTMS-NNToid group (Figure 5C-D). Excitatory signals, as indicated by cFOS expression, were transmitted to spinal interneurons (Figure 5E), subsequently reaching the I/G site and inducing cFOS expression in transplanted neurons (Figure 5F). cFOS-positive motor neurons were also observed in the L4 segment (Figure 5G), indicating that NNToid played a role in relaying excitatory neural information in the brain-spinal nerve pathway. To further investigate the reconstruction of spinal motor pathways, we injected BDA into the SMC region to anterogradely label CST axons. In the RN region, descending CST axon terminals were observed to contact the cell bodies of 5-HT neuron (Figure 5H-J). The rTMS-NNToid group exhibited significantly more BDA^+^ puncta on the surface of 5-HT neurons compared to the Sham-NNToid group (Figure 5L). The expression of the synaptic marker was detected at these contact sites (Figure 5M-N). Additionally, compared to the Sham-NNToid group, increased numbers of cFOS-positive cells were observed in the RN of the rTMS-NNToid group (Figure 5K, Figure S9), with no significant difference compared to the Normal group. These results suggested that rTMS-mediated excitation of layer V pyramidal neurons further activated the descending motor pathway, potentially enhancing neural regeneration and synapse formation.

### rTMS promotes CST and 5-HT fiber regeneration following NNToid transplantation

**Figure 6:**
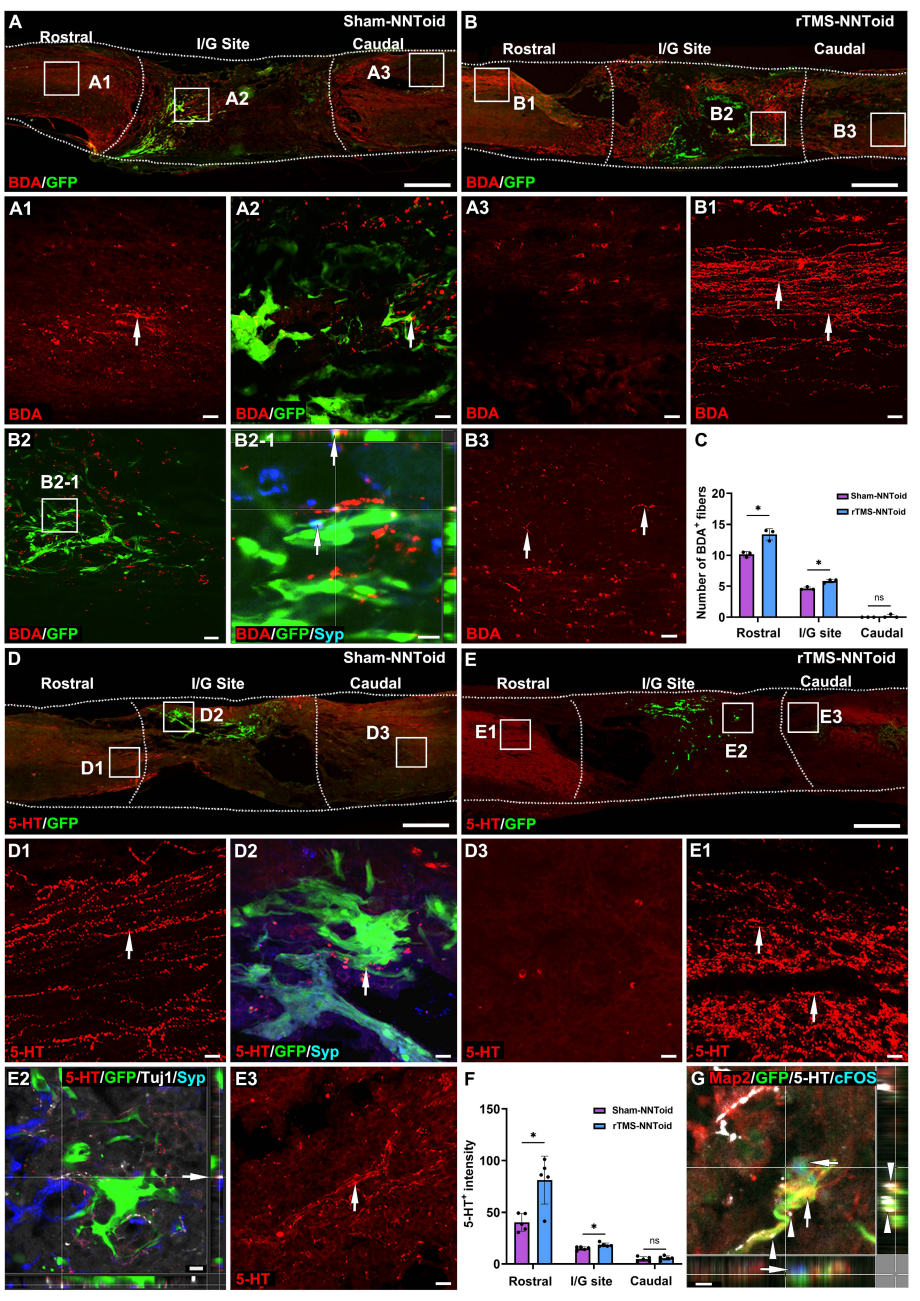
** rTMS-NNToid combination promoted regeneration of CST and 5-HT^+^ nerve fibers.** (A-B) The regeneration of the CST nerve fiber (arrows) in sagittal section of spinal cord in the Sham-NNToid and rTMS-NNToid groups 8 weeks post SCI. (A1-A3, B1-B3) The high magnification in the rostral, I/G site and caudal of the spinal cord. (B2-1) Orthogonal graph indicated the synaptic connections (arrows) between CST fibers and GFP NNToids. (C) Bar graph showing the number of BDA^+^ neural fibers (≥ 10 μm) in the rostral, I/G site and caudal of the injury area. *n* = 3. (D-E) The regeneration of 5-HT^+^ neural fibers (arrows) in sagittal section of spinal cord in Sham-NNToid and rTMS-NNToid groups. (D1-D3, E1-E3) The high magnification in the rostral, I/G site and caudal of the spinal cord. (E2) The regenerated 5-HT^+^ neural fibers in I/G site formed synaptic connections (arrows) with GFP NNToid neurons. (F) Bar graph showing the fluorescence density of 5-HT^+^ neural fibers in the rostral, I/G site and caudal of the injury area. *n* = 5. (G) Orthogonal view of the tight connection (arrows) between 5-HT^+^ neural fibers and cFOS^+^ excitatory GFP NNToid neurons in rTMS-NNToid group. I/G: injury/graft. Scale bars = 500 μm in (A-E), 40 μm in (D1-D3, E1, E3); 10 μm in (B2-1, E2). 5 μm in G. *p < 0.05, ns: non-significant. Data showed mean ± SD.

In the Sham-NNToid group, only limited CST fibers regeneration was observed in the rostral of the injury site (Figure 6A). Strikingly, the rTMS-NNToid group demonstrated substantially enhanced CST axonal regrowth in the rostral region compared to Sham-NNToid controls (Figure 6B). Additionally, increased CST fibers formed synaptic connections (Figure 6B2-1) with NNToid in the I/G site. And a small number of discontinuous short CST fibers were also observed in the caudal of the injury site. Quantitative analysis showed a significant increase in the number of CST fibers in the rostral and central regions compared to the Sham-NNToid group (Figure 6C). Furthermore, the rTMS-NNToid group enhanced the number of 5-HT^+^ fibers compared to the Sham-NNToid group at 8 weeks post SCI (Figure 6D-E). A greater number of 5-HT^+^ fibers regenerated into the I/G site (Figure 6F) and formed synapses with transplanted neurons within NNToid (Figure 6E2). This synaptic integration allowed the transplanted neurons to be excited by 5-HT neurotransmitters, leading to the expression of cFOS (Figure 6G).

### rTMS promotes the functional integration of the NNToid implant with host tissue

**Figure 7:**
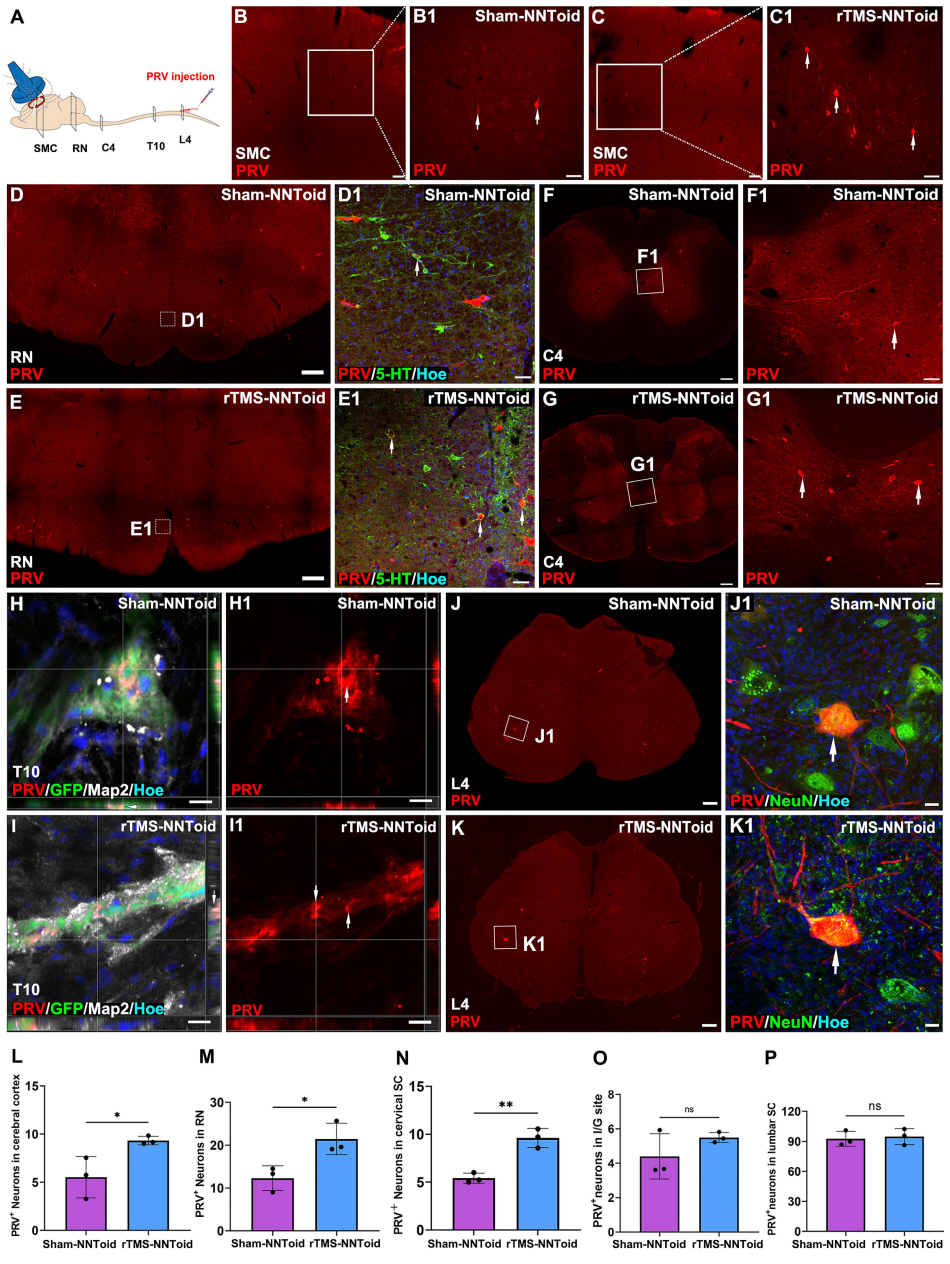
** Assessment of PRV^+^ neurons distribution.** (A) Diagram illustrating PRV retrograde transmission and the injection location (sciatic nerve). (B-C) Lower magnification of cross-section of SMC in the Sham-NNToid and rTMS-NNToid groups. (B1-C1) Higher magnification of PRV^+^ neurons (arrows) in the SMC region. (D-E) Representative images showing PRV^+^ neuron in mesencephalon of the Sham-NNToid and rTMS-NNToid groups. (D1-E1) Higher magnification of PRV^+^ neurons (arrows) in the RN. (F-G) Representative images showing PRV^+^ neurons in C4 spinal cord. (F1-G1) Higher magnification views of the PRV^+^ neurons (arrows) in (F-G). (H-I) Representative orthogonal images showing GFP^+^PRV^+^ neurons (arrows) in I/G site. (H1-I1) Representative images showing PRV^+^ neuron in I/G site. I/G: injury/graft. (J-K) Representative images showing PRV^+^ neurons in L4 spinal cord. (J1-K1) PRV^+^ neurons (arrows) in (J-K) are shown at higher magnification. (L-P) The bar chart showed the number of PRV^+^ neurons in SMC region, RN region, C4, I/G site, and L4. *n* = 3. SMC: Somatosensory motor cortex. RN: raphe nuclei, C4: cervical 4 segment, L4: lumbar 4 segment. Scale bars = 100 μm in (B-C), 50 μm in (B1-C1, D1-E1, F1-G1), 500 μm in (D-E), 200 μm in (F-G, J-K), 50 μm in (F1-G1), 10 μm in (H-I, H1-I1), 20 μm in (J1-K1). **p < 0.01, *p < 0.05, ns: non-significant. Data showed mean ± SD.

To investigate whether rTMS facilitate the integration of transplanted NNToid with the host spinal cord neuronal networks, PRV was injected into the bilateral sciatic nerves to achieve reverse transsynaptic tracing (Figure 7A) at 8 weeks post SCI. Surprisingly, PRV-labeled neurons were first observed in the SMC region (Figure 7B-C). And compared with the Sham-NNToid group (Figure 7B1), the number of PRV^+^ neurons of the rTMS-NNToid group (Figure 7C1) was significantly increased (Figure 7L). This observation suggested that the brain-spinal cord descending motor pathway was reconstructed, and rTMS could enhance the reconstruction. Furthermore, the efficacy of descending mesencephalon-spinal pathway was also modulated. A large number of PRV^+^ neurons in the RN region (Figure 7D-E), and the number of PRV^+^ neurons in the rTMS-NNToid group was significantly higher than that in the Sham-NNToid group (Figure 7M). We further identified 5-HT/PRV co-localized neurons, predominantly within RN of descending projections (Figure 7D1-E1). In the C4 segment above the injury plane, the rTMS-NNToid group also showed a significant increase in the number of PRV^+^ neurons compared to the Sham-NNToid group (Figure 7F-G, N), further supporting the long-distance reconstruction across the injury plane. In the I/G site, neurons differentiated from NNToid were infected by PRV (Figure 7H-I), indicating that the exogenous neurons received synaptic input from the host spinal cord neurons. However, no significant difference was detected in the number of PRV^+^ neurons between the Sham-NNToid and rTMS-NNToid groups at the I/G site (Figure 7O). The PRV-infected neurons at the L4 segment also did not differ significantly (Figure 7J-K, P).

### rTMS enhances the responsiveness of NNToid neurons to excitatory neural information

**Figure 8:**
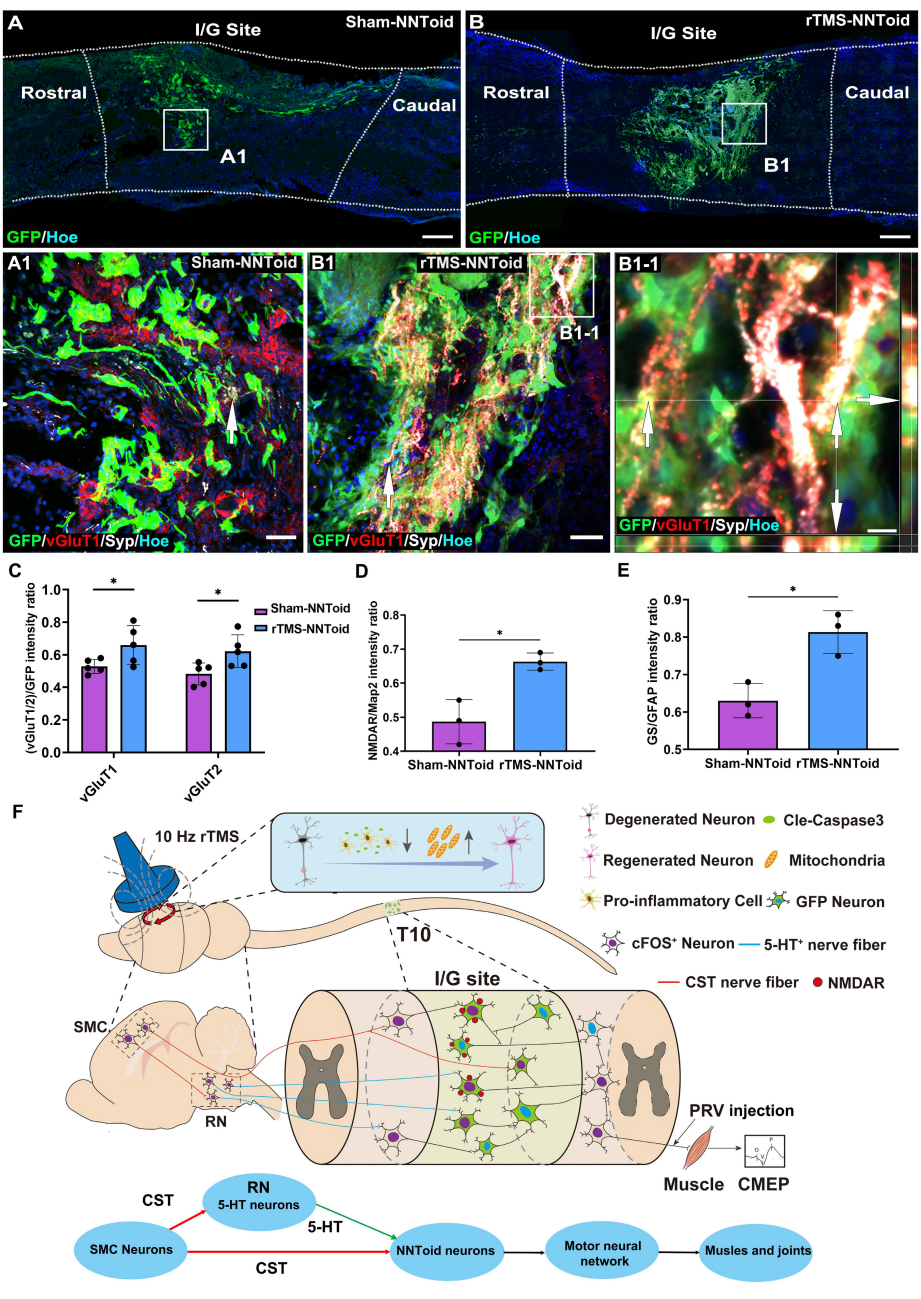
** rTMS and NNToid synergistically regulated excitatory information transmission.** (A-B) Low magnification images of the sagittal sections of the spinal cord in the Sham-NNToid group and rTMS-NNToid group. (A1-B1) The magnified areas showed the distribution of vGluT1 and Syp (arrows) on the surface of GFP NNToid and surrounding cells in the I/G site of Sham-NNToid and rTMS-NNToid groups. (B1-1) An orthogonal view showing vGluT1 and Syp double-positive GFP NNToid (arrows). (C) Bar graph showing the fluorescence density ratio of vGluT1/GFP and vGluT2/GFP in I/G site of the Sham-NNToid and rTMS-NNToid groups. *n* = 5. (D-E) Bar graph showing the fluorescence density ratio of NMDAR/Map2 and GS/GFAP in I/G site of the Sham-NNToid and rTMS-NNToid groups. *n* = 3. I/G: injury/graft. (F) Schematic diagram illustrating that rTMS-NNToids combination activated brain-spinal nerve pathway and promoted the regeneration of CST and 5-HT^+^ nerve fibers, further improved structurally integration ability of NNToid and functionally motor function of the hindlimb. Scale bars = 250 μm in (A-B); 50 μm in (A1-B1); 10 μm in (B1-1). *p < 0.05. Data showed mean ± SD.

The vGluT1 and vGluT2, which are responsible for packaging the major excitatory neurotransmitter glutamate into synaptic vesicles, serve as definitive markers for descending CST and excitatory interneurons. Immunofluorescence analysis exhibited extensive expression of vGluT1/2 and Syp at the I/G site in the rTMS-NNToid group compared to the Sham-NNToid group at 8 weeks post SCI (Figure 8A-B, Figure S10). In the rTMS-NNToid group, the expression of vGluT1 and vGluT2 (Figure 8C) in the I/G site was significantly increased, and the vGluT1 or vGluT2 positive fibers established synaptic connections with the NNToid (Figure 8B1-1 and Figure S10B1). Under the plasticity modulation effects of continuous rTMS treatment, the regenerated excitatory nerve fibers formed numerous synapses with the NNToid neurons, releasing glutamate to activate the transplanted neurons. In response to rTMS, transplanted neurons within NNToid upregulated the expression of N-methyl-d-aspartate receptors (NMDAR (Figure S11A, B and Figure 8D), enhancing their capacity to respond to and relay excitatory neural information. Concurrently, transplanted astrocytes within the NNToid exhibited increased the expression of glutamine synthetase (GS) (Figure S11C, D and Figure 8E), which supported efficient neural information relaying and contributed to the stabilization of descending motor pathway.

## Discussion

The groundbreaking aspect of this study is the synergistic combination of NNToid transplantation at the SCI site with rTMS, establishing a novel biophysical therapy paradigm. Notably, rTMS-induced upregulation of cFOS in SMC neurons not only reduced apoptosis but also activated the expression of gene collectively reactivating the intrinsic regenerative program. Most strikingly, our intervention enhanced CST-5-HT connectivity in the mesencephalon and enabled robust regeneration and functional integration of supraspinal fibers (CST/5-HT) within the NNToid graft (Figure 8F). PRV tracing confirmed that, under rTMS, NNToid more efficiently transmitted motor signals from the brain to hindlimb motor neurons, challenging the traditional view that brain-derived signals cannot reach paralyzed limbs after complete spinal cord transection.

### rTMS reverses SMC neuro-apoptosis and initiates nerve regeneration

Following complete SCI, cortical layer V pyramidal neurons undergo both axonal transection and loss of sensory feedback 32. Accumulating evidence indicated that the functional silencing of SMC neurons following acute SCI is a key contributor to neuroinflammation, neuronal apoptosis, and pathological structural remodeling 33-35. Imaging data revealed significant SMC atrophy by 40 days post-injury, underscoring the necessity for early neural stimulation therapy 36. We implemented a 10 Hz rTMS protocol initiated 72 hours post-injury. RNA-seq analysis revealed that rTMS upregulated genes associated with anti-inflammatory and anti-apoptotic pathways. Histological findings confirmed a significant reduction in microglial activation and apoptosis levels in SMC neurons after 4 weeks of treatment. These findings suggest that rTMS effectively alleviated neuroinflammation in the SMC region, thereby promoting the survival of cortical motor neurons and enhancing axonal regeneration. However, there is currently limited evidence from comparative studies to guide the optimal selection of treatment parameters.

Several studies have indicated that rTMS frequencies exceeding 20 Hz can induce more extensive neuronal excitation in the SMC 37-39. Our results suggest that 10 Hz rTMS is advantageous for modulating descending cortical pathways by preferentially exciting layer V pyramidal neurons. This stimulation paradigm preferentially activates these neurons, enhancing the expression of mitochondrial-related proteins TOM20, NF and synaptic-related proteins Syp. We propose that these effects are crucial for initiating regenerative processes, including cytoskeletal protein synthesis and neuroplasticity. Transcriptomic analysis indicated that rTMS induced upregulation of genes associated with neuronal activation, regeneration, and synaptic plasticity. Following 4 weeks of rTMS treatment, the expression levels of p-S6 and Arc in the SMC—key markers of axonal regeneration and synaptic plasticity 31,40—were significantly upregulated in layer V pyramidal neurons. These findings suggest that rTMS initiates the regeneration of cortical motor neurons through neuronal activation, thereby exerting sustained or potentially long-lasting effects on neural regeneration and plasticity. This may account for why long-term rTMS treatment outperforms transient brain stimulation effects. Another critical challenge lies in guiding the axons of regenerated motor cortex neurons to locate target neurons at the injury site. Otherwise, the regenerated axons will be at risk of retraction.

### NNToid transplantation facilitates host nerve fiber regeneration and functional integration

Although rTMS increases the number of brain-derived neural fibers regenerating into the SCI region, in the rat SCI model with 2 mm of spinal cord tissue removed, it remains challenging for these fibers to regenerate across the injury site and reach the caudal end. In the absence of timely supplementation of new neurons, the axons that have regenerated into the injury site may undergo retraction.

Compared to strategies such as transplanting NSCs or recruiting endogenous NSCs, NNToid transplantation offers several distinctive advantages: 1. Overexpression of *NT-3* creates a favorable microenvironment for NNToid survival, host nerve regeneration, and synapse formation 41. 2. The inclusion of neurons overexpressing *TrkC* enhances the attraction of CST axons that express receptor protein tyrosine phosphatase sigma (PTPσ) 42,43. 3. Predominant differentiation into excitatory neurons with robust expression of synaptic proteins indicates a higher potential for these neurons to respond to neural plasticity regulation 44. 4. The presence of a small number of oligodendrocytes and astrocytes further supports axonal myelination 45 and enables responsiveness to rTMS-induced synaptic plasticity regulation within the NNToid. However, current research primarily focuses on rTMS-induced modulation of brain neural circuits 17. Whether rTMS can regulate neural network plasticity following NNToid transplantation remains to be further elucidated.

### rTMS modulates NNToid-mediated relaying of excitatory neural signals

To achieve sustained functional recovery following SCI, rTMS-mediated neuroplasticity must facilitate the establishment of beneficial motor pathways^46^,^47^. We investigated the effects of rTMS on CST-controlled motor pathways following NNToid transplantation. Our findings demonstrate that rTMS-induced excitation of layer V pyramidal neurons enhances excitatory signaling cascades across multiple neural substrates, including serotonergic neurons within the mesencephalon RN, T1 spinal cord long descending propriospinal neurons, NNToid graft neurons, and L4 spinal cord motor neurons. We propose a dual mechanism for rTMS in modulation of NNToid neurons: direct activation via regenerated CST terminals and indirect excitation through descending intrinsic fibers from the brainstem and cervical spinal cord. This dual activation pathway enables NNToid neurons to relay excitatory signals to motor neurons controlling hindlimb muscle.

Experimental validation revealed enhanced CST growth into RN, with synaptic integration into the 5-HT^+^ neurons. Additionally, more CST and 5-HT neural fibers regenerated and grew into the NNToid grafts following rTMS treatment. These results indicate that rTMS facilitates stable synaptic integration between NNToid neurons and both CST and 5-HT nerve fibers, thereby establishing long-term plasticity that may sustain voluntary movement independently of continued stimulation. This mechanism parallels the effects of deep brain stimulation (DBS) in incomplete SCI models but differs in its cortical approach via SMC stimulation, rather than direct electrode stimulation in the mesencephalon or thalamic 48,49. The cortical integration of excitatory signals via layer V pyramidal neurons enables more extensive activation of brainstem motor nuclei, thereby approaching the motor control mode under physiological conditions. Notably, another key difference is the animal model we utilized, which involves a 2 mm spinal cord transection. This implies that all excitatory neural signals induced by rTMS are transmitted exclusively via NNToid neurons, rather than through residual neural fibers. Furthermore, our study emphasizes the long-term neuroplasticity effects of stimulation, as opposed to its transient effects. One limitation of the present study lies in the lack of validation through large animal models, such as canines and non-human primates. Additionally, another limitation is the absence of additional motor rehabilitation training. This training might have further enhanced functional recovery by preventing disuse atrophy.

After 8 weeks of rTMS treatment, NNToid neurons exhibited more extensive integration with CST and 5-HT terminals. Additionally, a denser network of glutamatergic excitatory fibers formed around these neurons, leading to the establishment of functional synaptic connections. PRV tracing after sciatic nerve injection confirmed that more neurons in the RN and SMC regions were labeled via trans-synaptic transmission through NNToid neurons. The glutamate receptor NMDAR was upregulated in NNToid neurons, enhancing their responsiveness to and transmission of excitatory neural information. Concurrently, NNToid astrocytes stabilize the established neural information relay pathway through increased expression of glutamine synthetase. The rTMS-induced alterations collectively contribute to the establishment of a stable descending motor pathway mediated by NNToid neurons.

## Conclusion

rTMS treatment effectively inhibits cortical motor neuron apoptosis and promotes CST regeneration and plasticity modulation of CST-controlled motor pathways following NNToid transplantation. NNToid neurons promote the ingrowth of brain-derived descending fibers into the transplant site and act as target cells that relay excitatory signals and respond strongly to sustained rTMS stimulation, thereby stabilizing the corticobulbar-NNToid neuron-lumbar spinal cord motor neuron pathway. The synergistic effect between NNToid neurons and rTMS offers new insights for combined physical and biological therapies in treating central nervous system injuries. However, this study did not employ techniques such as monosynaptic tracing and single-cell spatial transcriptomics to identify NNToid neuron subtypes responsive to rTMS modulation. Future research should incorporate advanced spatial transcriptomic and metabolomic approaches to address this limitation and investigate treatment effects over extended time points. Furthermore, combined lumbosacral nerve stimulation may be explored as a therapeutic strategy, and the clinical translational potential of autologous induced pluripotent stem cell-derived NNToid transplantation warrants evaluation.

## Supplementary Material

Supplementary figures and table.

Supplementary movie 1.

Supplementary movie 2.

Supplementary movie 3.

## Abbreviations

NSCs: neural stem cells
SMC: sensorimotor cortex
rTMS: repetitive transcranial magnetic stimulation
iTBS: intermittent theta-burst stimulation
NNToid: neural network tissueoid
AAV: adeno-associated virus
PLL: polylysine
BBB: Basso-Beattie-Bresnahan
CMEP: Cortical Motor Evoked Potentials
BDA: biotinylated dextran amine
PFA: paraformaldehyde
PVDF: polyvinylidene fluoride
TBST: Tris-buffered saline containing Tween-20
ECL: enhanced chemiluminescence
PBS: phosphate-buffered saline
CST: corticospinal tract
5-HT: 5-hydroxytryptamine
Arc: activity regulated cytoskeletal
and TOM20: translocase of outer mitochondrial membrane 20
CC3: cleaved caspase 3
IBA-1: ionized calcium-binding adapter molecule-1
p-S6: phosphorylated S6
GAP43: growth-associated protein 43
Tuj1: β-tubulin III
NeuN: neuronal nuclear antigen
Olig2: oligodendrocyte precursor transcription factor 2
Map2: microtubule-associated protein 2
Syp: synaptophysin
PSD95: postsynaptic density protein 95
PRV: pseudorabies virus
vGluT1: vesicle glutamate transporters 1
vGluT2: vesicle glutamate transporters 2
GAD67: glutamate decarboxylase 67
I/G: Injury/Graft
NMDAR: N-methyl-d-aspartate receptor
GS: glutamine synthetase
RN: raphe nuclei
LDPT: long descending propriospinal tract
DBS: deep brain stimulation
PPI: protein-protein interaction
HE: hematoxylin and eosin
