# Supplementary material for: rTMS-induced motor cortex activation drives neural network tissueoid mediated spinal motor neural pathway reconstruction
Source: Theranostics. 2026 Jan 1;16(2):915–35. doi: 10.7150/thno.117789 (PMC12674999; doi:10.7150/thno.117789)
Supplement: Supplementary file 1 — Supplementary figures and table. [file thnov16p0915s1.pdf]

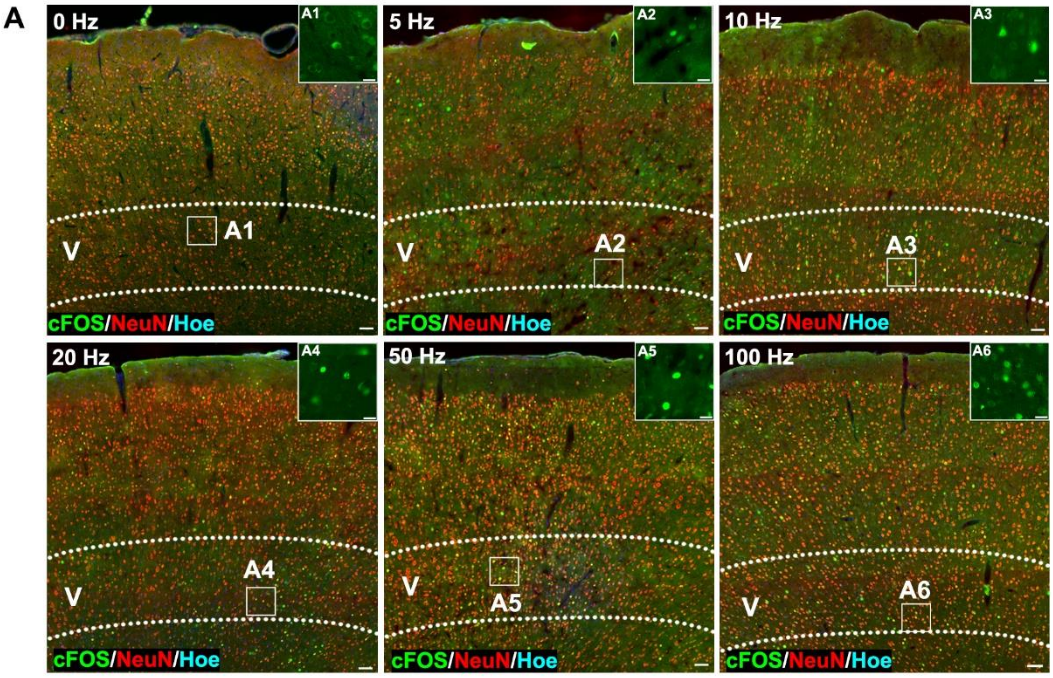

Figure S1. The expression range of cFOS in SMC neurons under rTMS at various frequencies.

(A) Representative images showed the expression of cFOS in the SMC neurons of the Normal rats under 0, 5, 10, 20, 50 and 100 Hz rTMS stimulation. (A1-A6) The high magnification views correspond to the cFOS expression in the layer V neurons. Scale bars = 60 μm in A, 20 μm in (A1-A6).

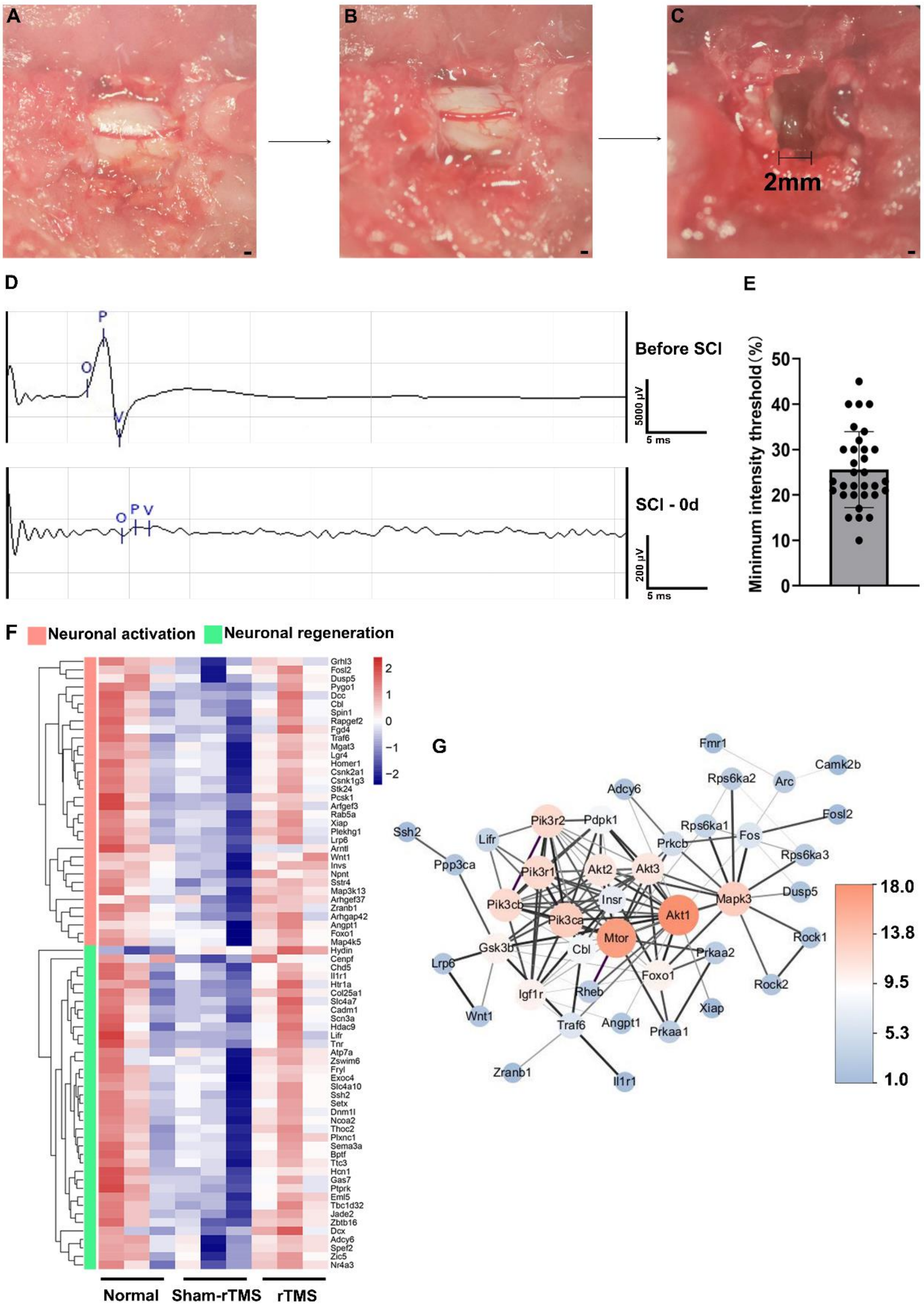

Figure S2. rTMS induced neuronal activation and regeneration.

(A) Representative dorsal view of the intact spinal cord prior to transection. (B) Removal of the spinal dura mater exposing the spinal cord surface. (C) Representative dorsal view of the spinal cord following complete transection at the T10 level. (D)

Assessment of CMEPs before and after complete T10 spinal cord transection in the rats. (E) Bar graph illustrating the minimal rTMS stimulation intensity required to elicit hindlimb motor evoked potentials in naïve rat. *n* = 32. (F) A heatmap was generated based on significantly differentially expressed up-regulated and down-regulated genes related to neuronal activation, neuronal regeneration in the SMC region 4 weeks post SCI. (G) The PPI network of 41 genes related to neural activation, regeneration and synaptic plasticity pathways. Each node represents a protein，the size and color are scaled to its degree centrality. The edges indicated protein-protein interactions, and line thickness indicated the strength of data support. PPI：protein-protein interaction network. Scale bars = 500 μm in (A-C).

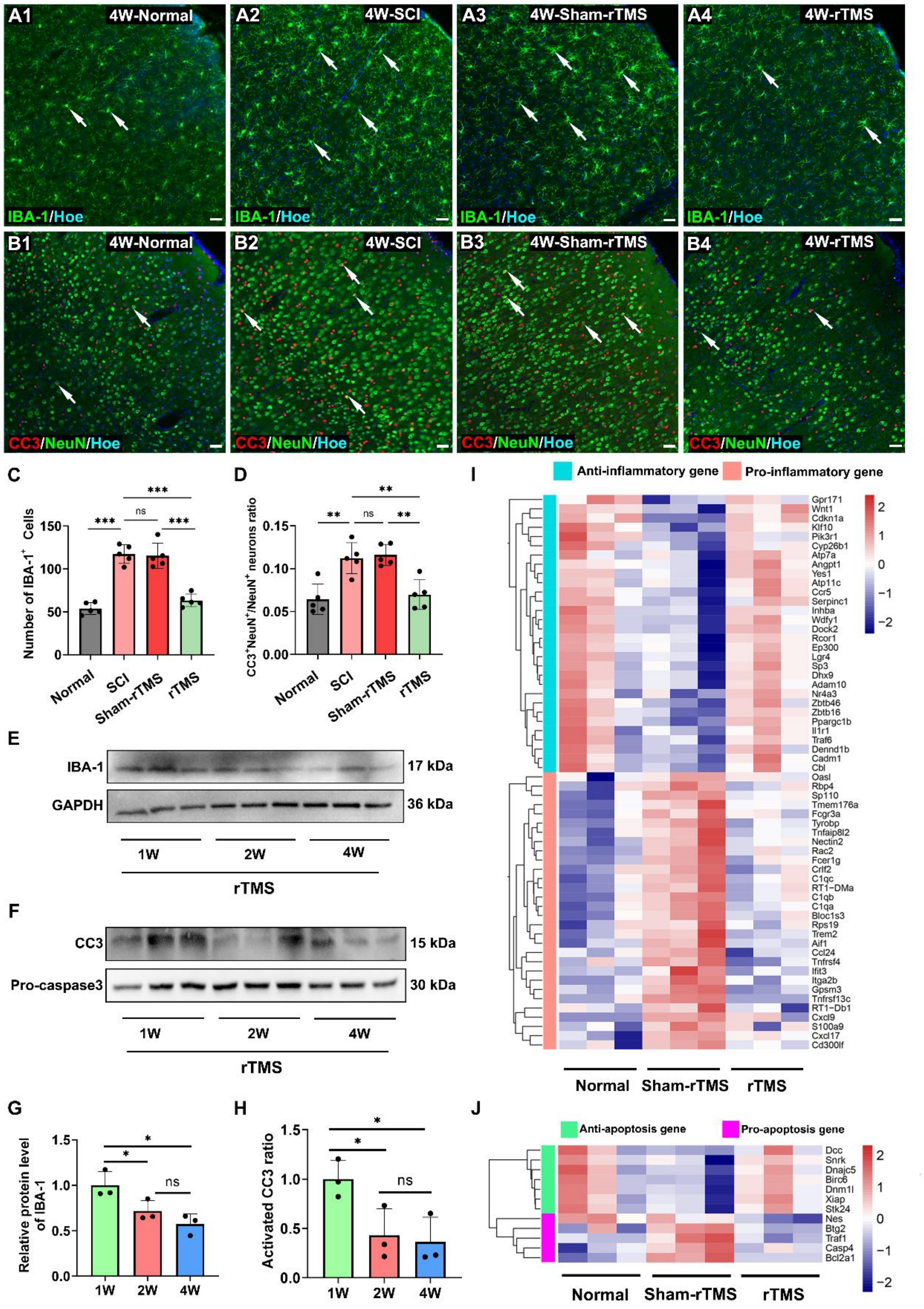

Figure S3. rTMS inhibited inflammation and neuronal apoptosis in SMC region.

(A) Immunofluorescent representation of IBA-1 in SMC region 4 weeks post SCI, and arrows indicated typical IBA-1^{+} microglia. (B) Immunofluorescent representation of

$CC3^+$ $NeuN^+$ neurons (arrows) in SMC region 4 weeks post SCI. (C-D) Quantification of IBA-1 and CC3 expressions. $n = 5$. (E-F) Western blots revealed the expression of IBA-1 and CC3 proteins in SMC region at 1, 2 and 4 weeks post SCI in rTMS group. (G- H) Quantitative analysis of IBA-1 and CC3 proteins expression. $n = 3$. (I-J) Heatmap was constructed from the up-regulated and down-regulated genes with significant differential expression relevant to inflammation and apoptosis in SMC region of Normal, Sham-rTMS and rTMS groups. $n = 3$. Scale bars = 40 μm in (A-B). $***p < 0.001$, $**p < 0.01$, $*p < 0.05$, ns: non-significant. Data showed mean ± SD.

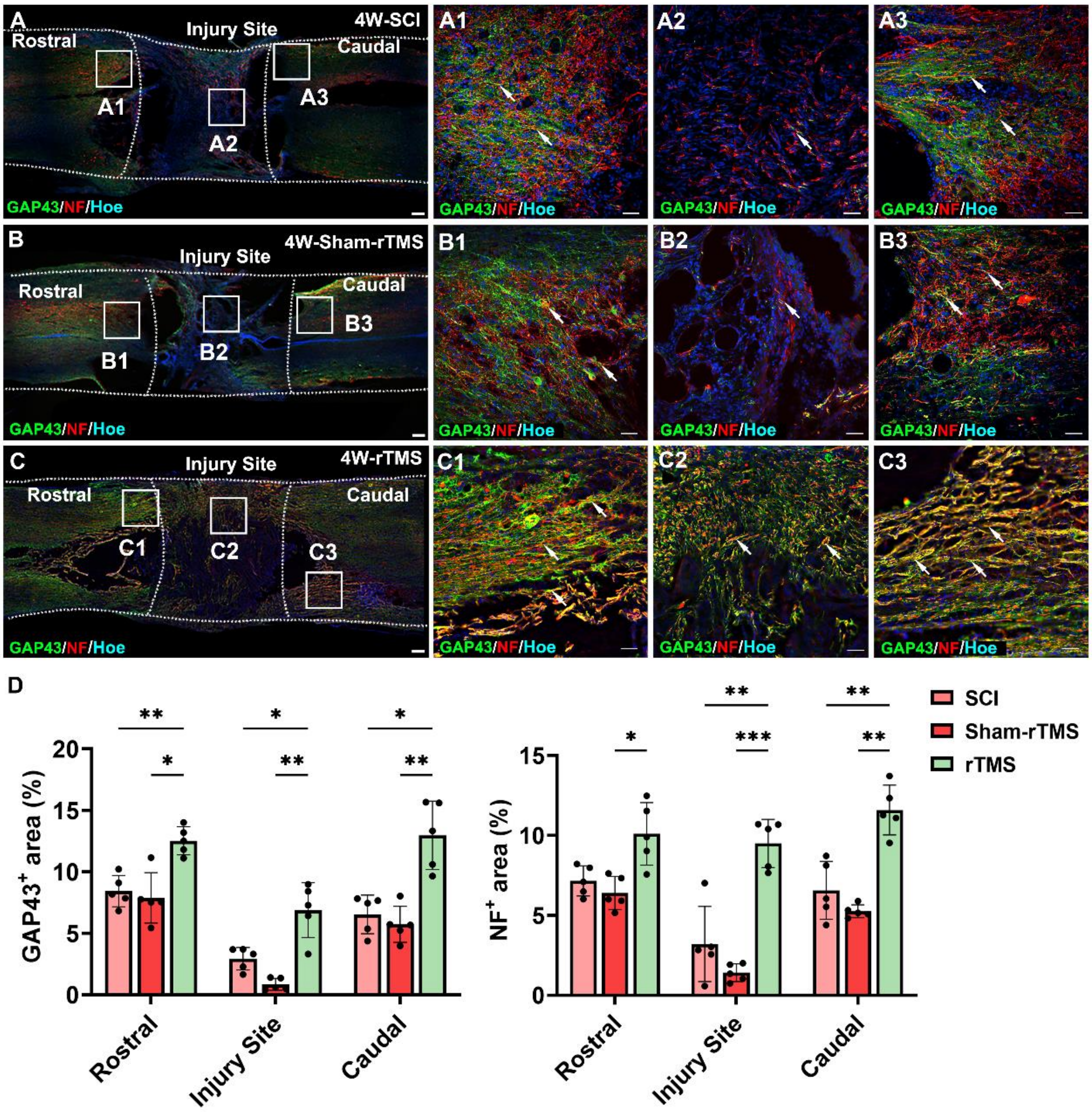

Figure S4. rTMS promoted nerve fiber regeneration.

(A-C) Low-power microscopic views of spinal cord sagittal sections in the SCI, Sham-rTMS, and rTMS group 4 weeks post SCI. (A1-A3, B1-B3, C1-C3) indicated the rostral, central, caudal of the injured area, respectively. Arrows indicated $GAP43^+$ NF^+ fibers. (D) Bar graphs showed the fluorescence area ratio of $GAP43^+$ fibers and NF^+ fibers. $n = 5$. Scale bars = 250 μm in (A-C), 50 μm in (A1-C1, A2-C2, A3-C3). $***p < 0.001$, $**p < 0.01$, $*p < 0.05$. Data showed mean ± SD.

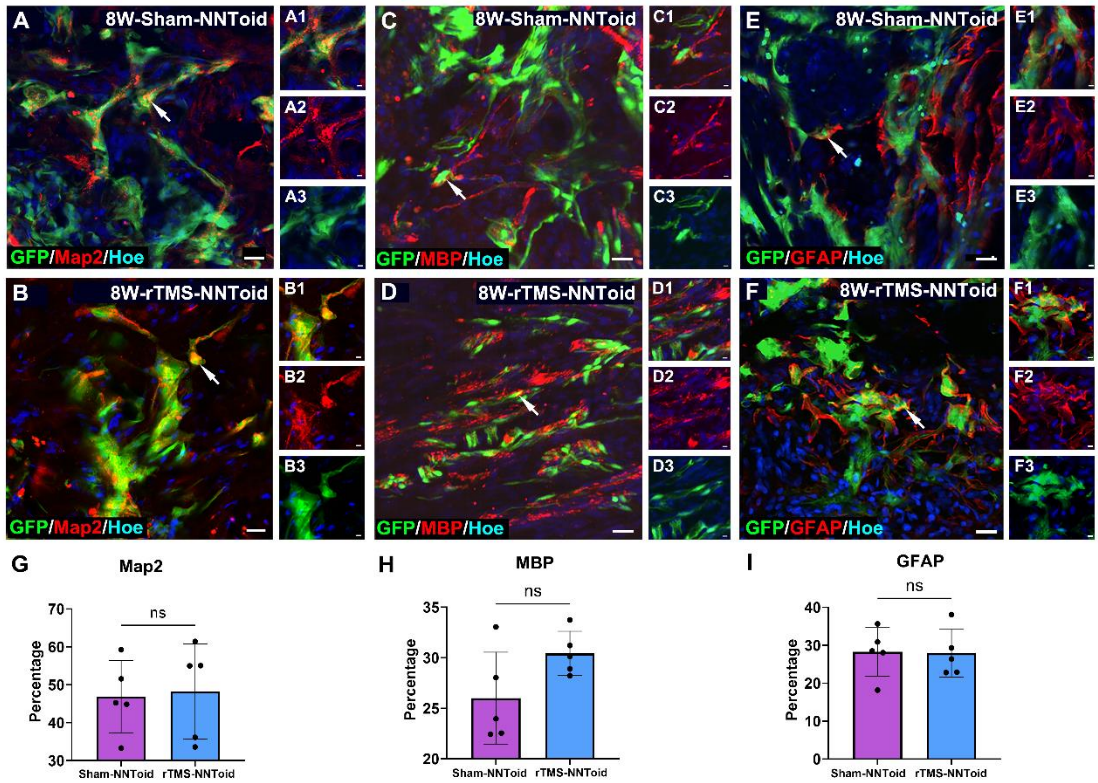

Figure S5. The differentiation within the NNToid in vivo.

(A-B) The GFP$^+$/Map2$^+$ (arrows), (C-D) GFP$^+$/MBP$^+$ (arrows) and (E-F) GFP$^+$/GFAP$^+$ cells (arrows) derived from GFP$^+$ transplanted cells within NNToid in the I/G site of Sham-NNToid and rTMS-NNToid groups. (A1-F1, A2-F2, A3-F3) The local images showed the typical GFP$^+$/Map2$^+$, GFP$^+$/MBP$^+$, and GFP$^+$/GFAP$^+$ cells in (A-F), respectively. I/G: Injury/Graft. (G-I) The percentages of GFP$^+$/Map2$^+$, GFP$^+$/MBP$^+$, and GFP$^+$/GFAP$^+$cells in the number of GFP$^+$ cells, respectively. n = 5. Scale bars = 20 µm in (A-F), 5 µm in (A1-F1, A2-F2, A3-F3). ns: non-significant. Data showed mean ± SD.

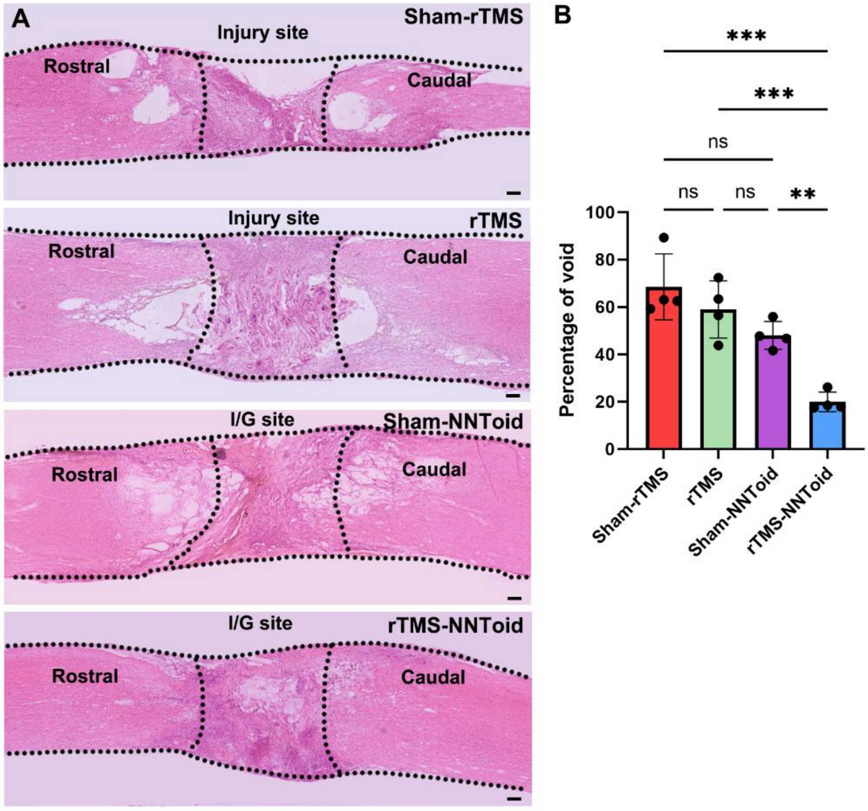

Figure S6. Morphological analysis of spinal cord tissue at 8-week post-injury.

(A) Representative HE-stained images of spinal cord tissue of the Sham-rTMS, rTMS, Sham-NNToid, and rTMS-NNToid groups in sagittal sections. (B) Quantification of the void ratio within the I/G site. $n = 4$. ***$p < 0.001$, **$p < 0.01$, ns: non-significant. Data showed mean ± SD.

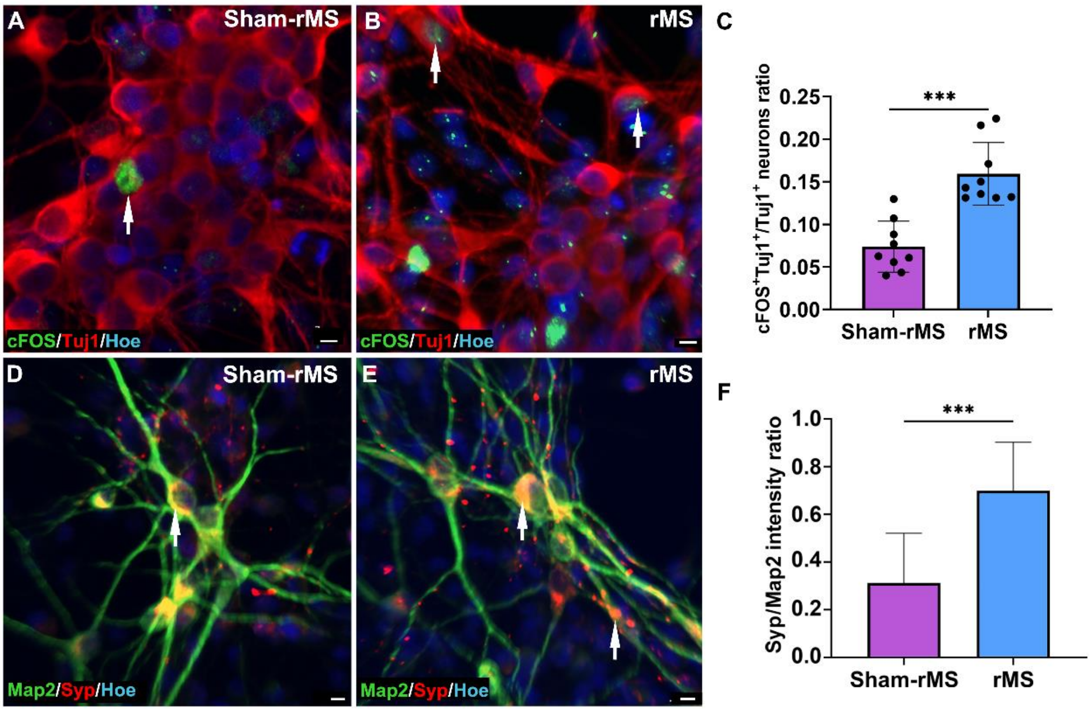

Figure S7. rMS activated SMC neurons in vitro.

(A-B) Immunofluorescence staining of cFOS (arrows) in SMC neurons after Sham-rMS and rMS in vitro. (C) Bar chart revealed the number ratio of cFOS^{+}Tuj1^{+}/Tuj1^{+} neurons in Sham-rMS and rMS groups. (D-E) Immunofluorescence staining of Syp (arrows) in SMC neurons after Sham-rMS and rMS in vitro. (F) Bar chart revealed the fluorescence intensity ratio of Syp/Map2 in Sham-rMS and rMS groups. Scale bars = 5 μm in (A-B, D-E). ***$p < 0.001$. Data showed mean ± SD.

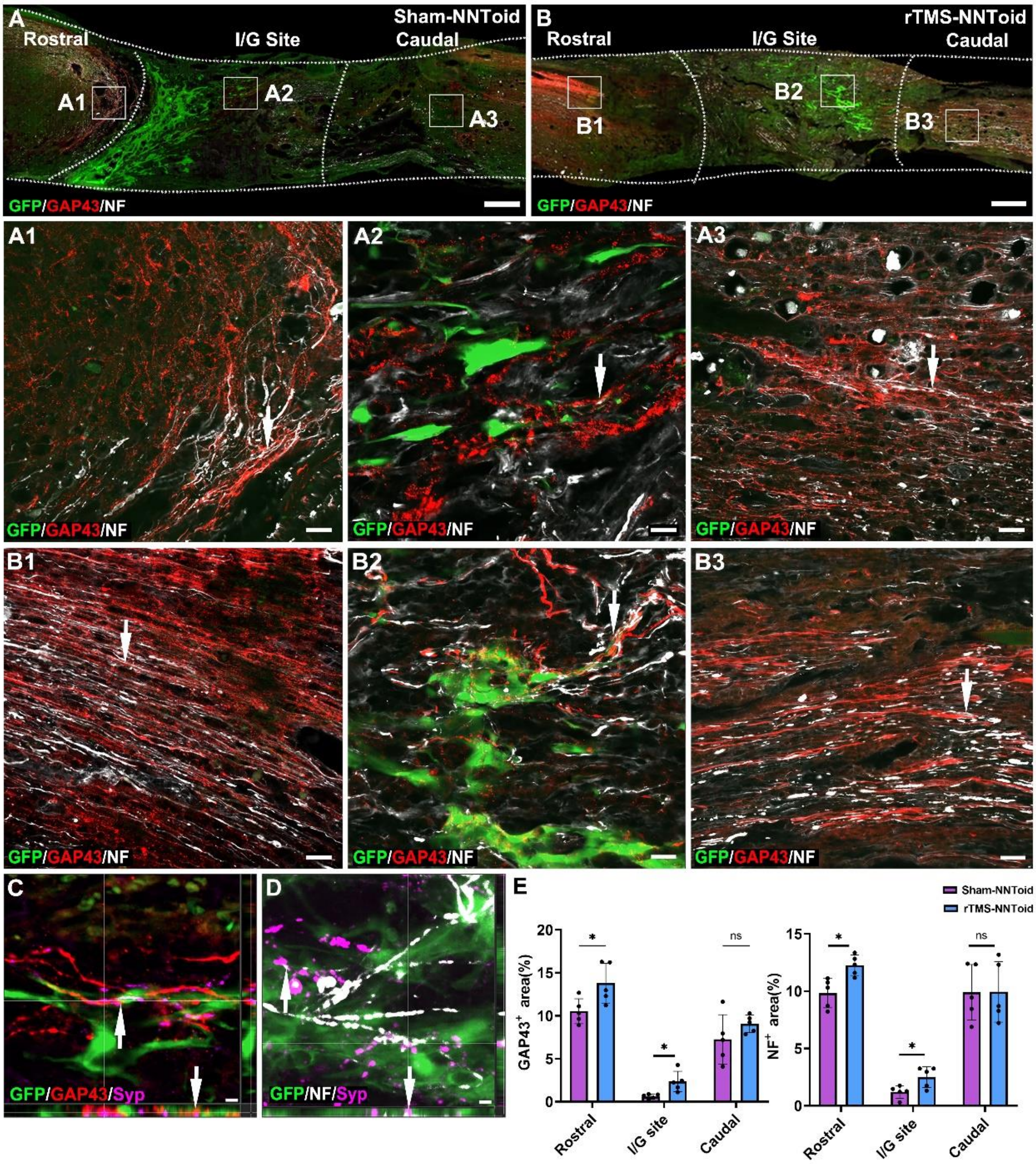

Figure S8. rTMS-NNToid combination promoted nerve fiber regeneration.

(A-B) Immunofluorescence expression of GAP43 and NF in sagittal section of spinal cord. (A1-A3, B1-B3) Plots represented high magnification views in the (A-B), arrows indicated GAP43 and NF double positive neural fibers. (C-D) Representative images demonstrate that GFP^+ cells in the I/G site express GAP43 or NF, and establish synaptic connections (arrows) with host neural fibers in rTMS-NNToid group. (E) Quantitative analysis showed average fluorescence area ratio of $GAP43^+$ and NF^+ neural fibers in

the rostral, I/G site and caudal of the injury area. n = 5. I/G: Injury/Graft. Scale bars = 400 μm in (A-B), 20 μm in (A1-A3, B1-B3); 5 μm in (C, D). *p < 0.05, ns: non-significant. Data showed mean ± SD.

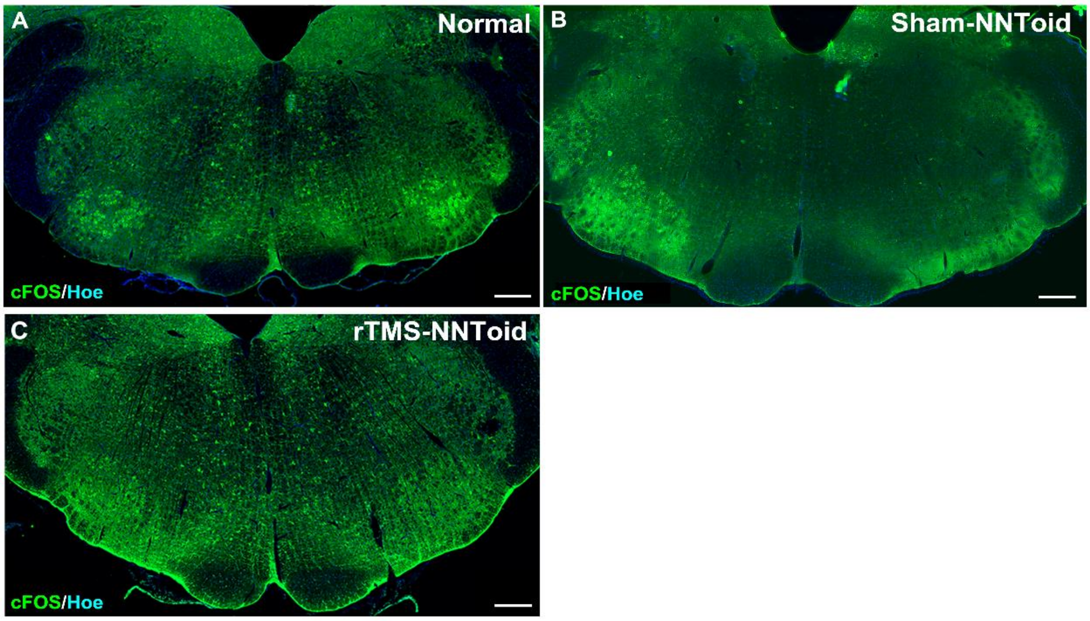

Figure S9. **Expression of cFOS in the RN of the Normal (A), Sham-NNToid (B), and rTMS-NNToid (C) groups**.

Scale bars = 500 μm.

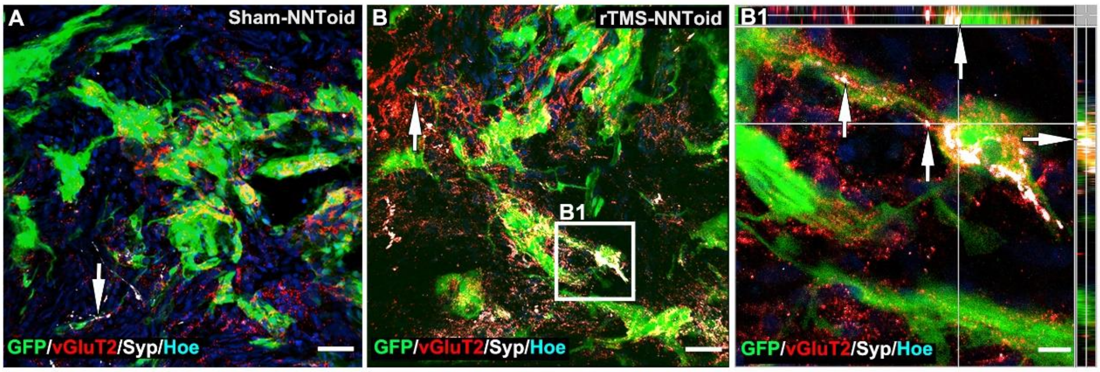

Figure S10. Expression of vGluT2 in the I/G site.

(A-B) Distribution of vGluT2 and Syp on the surface of GFP cells (arrows) and surrounding cells in the I/G site of Sham-NNToid and rTMS-NNToid groups. (B1) An

orthogonal view showing vGluT2 and Syp double-positive GFP NNToids (arrows). Scale bars = 50 μm in (A-B); 10 μm in (B1).

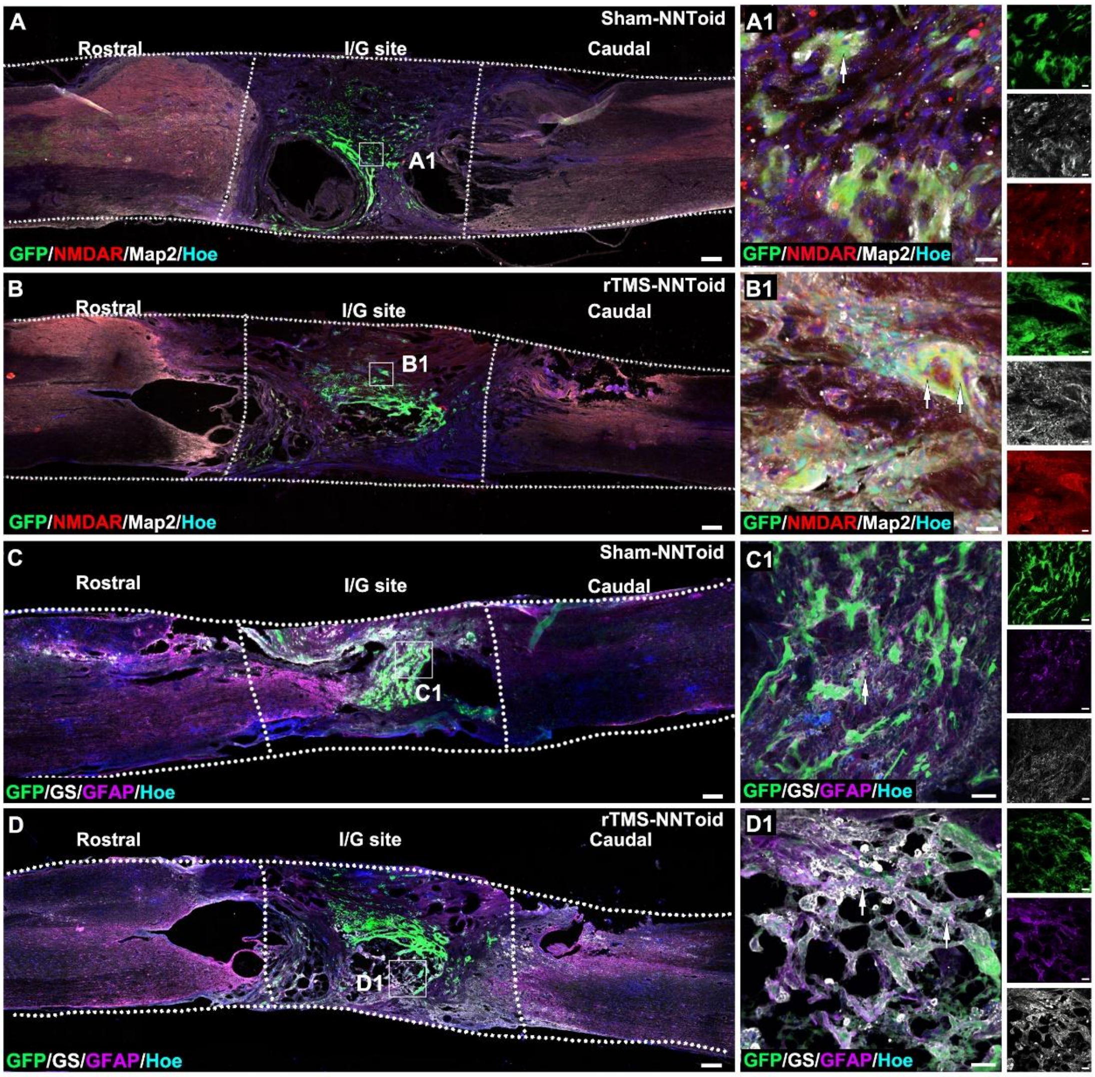

Figure S11. rTMS upregulated the expression of NMDAR and GS in the NNToid. (A-B) Immunofluorescence expression of NMDAR and Map2 in sagittal section of spinal cord in the Sham-NNToid group and the rTMS-NNToid group. (C-D) Immunofluorescence expression of GS and GFAP in sagittal section of spinal cord in the Sham-NNToid group and the rTMS-NNToid group. (A1-D1) Plots represented high magnification views in the I/G site of (A-D). Scale bars = 250 μm in (A-D), 20 μm in (A1-D1). Data showed mean ± SD.

Table S1. List of antibodies.

Antibodies	Tedia Company Inc	Dilution Rate
Map2 (Rabbit)	Cell signaling Technology (4542S, USA)	1:500 (IF)
Map2 (Mouse)	Sigma-Aldrich (M9942, USA)	1:1000 (IF)
MBP (Rabbit)	Abcam (ab40390, UK)	1:200 (IF)
GFAP (Rabbit)	Abcam (ab7260, UK)	1:500 (IF)
Avidin-647 (Rabbit)	invitrogen (S32357, USA)	1:500 (IF)
Syp-647 (Rabbit)	Abcam (ab196166, UK)	1:500 (IF)
NEUN-647 (Rabbit)	Cell signaling Technology (62994s, USA)	1:500 (IF)
NeuN (Rabbit)	Cell signaling Technology (12943S, USA)	1:200 (IF)
P-S6 (Rabbit)	Cell signaling Technology (5364T, China)	1:1000 (WB)<br>1:200 (IF)
Arc (Rabbit)	Abcam (ab183183, UK)	1:1000 (WB)<br>1:200 (IF)
vGluT1 (Mouse)	Snaptic Systems (135011BT, USA)	1:500 (IF)
vGluT2 (Rabbit)	Snaptic Systems (135403, USA)	1:500 (IF)
TOM20 (Mouse)	Santa Cruz Biotechnology (SC-1776, USA)	1:50 (IF)
NF (Rabbit)	Beyotime (AF1423, China)	1:200 (IF)
Nestin (Mouse)	Abcam (AB6142, UK)	1:500 (IF)
TrkC (Rabbit)	Cell signaling Technology (3376S, USA)	1:200 (IF)
Tubblin III (Mouse)	Abcam (ab78078, UK)	1:1000 (IF)

Tubblin III (Rabbit)	Sigma-Aldrich (T2200-200UL, USA)	1:1000 (IF)
NT3 (Rabbit)	BOSTER (BA1293, China)	1:200 (IF)
Olig2 (Rabbit)	Abcam (ab109186, UK)	1:1000 (IF)
PSD95 (Mouse)	Cell signaling Technology (36233S, USA)	1:500 (IF)
GAD67 (Rabbit)	Abcam (ab213508-1001, UK)	1:200 (IF)
GAP43 (Mouse)	Merck millipore (MAB347, Germany)	1:200 (IF)
c-FOS (Mouse)	Santa Cruz Biotechnology (SC-8047, USA)	1:100 (IF)
5-HT (Rabbit)	Sigma (S5545, US)	1:5000 (IF)
IBA-1 (Rabbit)	Wako (SKN4887, Japan)	1:2000 (WB)<br>1:400 (IF)
NMDAR (Rabbit)	Sigma (SAB4501301, US)	1:50 (IF)
Glutamine Synthetase (Rabbit)	Abcam (ab73593)	1:200 (IF)
Cle-caspase3 (Rabbit)	Cell signaling Technology (D175, US)	1:1000 (WB)<br>1:200 (IF)
Pro-caspase3 (Rabbit)	Cell signaling Technology (9662S, US)	1:1000 (WB)
GAPDH (Rabbit)	Sigma (SAB4300645, US)	1:3000 (WB)
β-Actin-HRP (Rabbit)	Beyotime (AF5006, China)	1:3000 (WB)
Alexa Fluor ® 488	Abcam (ab150077, UK)	1:500 (IF)

Alexa Fluor ® 555	Abcam (ab150169, UK)	1:500 (IF)
Alexa Fluor Ⓜ 488	Abcam (ab150113, UK)	1:500 (IF)
Alexa Fluor Ⓜ 555	Abcam (ab150114, UK)	1:500 (IF)
Alexa Fluor Ⓜ 647	Abcam (ab150115, UK)	1:500 (IF)
Hoechst33342	Beyotime (C1022, China)	1:500 (IF)
